# HLA Risk Alleles in Aromatic Antiepileptic Drug-Induced Maculopapular Exanthema

**DOI:** 10.3389/fphar.2021.671572

**Published:** 2021-05-26

**Authors:** Yi-Wu Shi, Jie Wang, Fu-Li Min, Wen-Jun Bian, Bi-Jun Mao, Yong Mao, Bing Qin, Bing-Mei Li, Yang-Mei Ou, Yun-Qi Hou, Xin Zou, Bao-Zhu Guan, Na He, Yong-Jun Chen, Xue-Lian Li, Juan Wang, Wei-Yi Deng, Han-Kui Liu, Nan-Xiang Shen, Xiao-Rong Liu, Yong-Hong Yi, Lie-Min Zhou, Dong Zhou, Patrick Kwan, Wei-Ping Liao

**Affiliations:** ^1^ Institute of Neuroscience and Department of Neurology of the Second Affiliated Hospital of Guangzhou Medical University; Key Laboratory of Neurogenetics and Channelopathies of Guangdong Province and the Ministry of Education of China, Guangzhou, China; ^2^ Department of Neurology, Guangzhou First People’s Hospital, Guangzhou, China; ^3^ BGI-Shenzhen, Shenzhen, China; ^4^ Epilepsy Center and Department of Neurosurgery, The First Affiliated Hospital, Jinan University, Guangzhou, China; ^5^ Department of Neurology, Guangdong 999 Brain Hospital, Guangzhou, China; ^6^ The First People’s Hospital of Shunde, Foshan, China; ^7^ The Third People’s Hospital of Mianyang, Mianyang, China; ^8^ Department of Neurology, Nanhua Hospital Affiliated to South China University, Hengyang, China; ^9^ Department of Neurology, The Affiliated Yuebei People’s Hospital of Shantou University Medical College, Shaoguan, China; ^10^ The Affiliated Hospital of Xiangnan University, Chenzhou, China; ^11^ Department of Neurology, The Seventh Affiliated Hospital, Sun Yat-Set University, Guangzhou, China; ^12^ West China Hospital, Sichuan University, Chengdu, China; ^13^ Department of Neuroscience, Central Clinical School, Monash University, Alfred Hospital, Melbourne, VIC, Australia

**Keywords:** antiepileptic drugs, oxcarbazepine, maculopapular exanthema, human leukocyte antigen, risk factor

## Abstract

To characterize human leukocyte antigen (HLA) loci as risk factors in aromatic antiepileptic drug-induced maculopapular exanthema (AED-MPE). A case-control study was performed to investigate HLA loci involved in AED-MPE in a southern Han Chinese population. Between January 2007 and June 2019, 267 patients with carbamazepine (CBZ), oxcarbazepine (OXC), or lamotrigine (LTG) associated MPE and 387 matched drug-tolerant controls from six centers were enrolled. HLA-A/B/C/DRB1 genotypes were determined using sequence-based typing. Potential risk alleles were validated by meta-analysis using data from different populations and *in silico* analysis of protein-drug interactions. HLA-DRB1*04:06 was significantly associated with OXC-MPE (*p* = 0.002, *p*_*c*_ = 0.04). HLA-B*38:02 was associated with CBZ-MPE (*p =* 0.03). When pooled, HLA-A*24:02, HLA-A*30:01, and HLA-B*35:01 additionally revealed significant association with AED-MPE. Logistic regression analysis showed a multiplicative interaction between HLA-A*24:02 and HLA-B*38:02 in CBZ-MPE. Meta-analysis of data from different populations revealed that HLA-24*:02 and HLA-A*30:01 were associated with AED-MPE (*p* = 0.02 and *p* = 0.04, respectively). *In silico* analysis of protein-drug interaction demonstrated that HLA-A*24:02 and HLA-A*30:01 had higher affinities with the three aromatic AEDs than the risk-free HLA-A allele. HLA-DRB1*04:06 showed relatively specific high affinity with S-monohydroxy derivative of OXC. HLA-DRB1*04:06 is a specific risk allele for OXC-induced MPE in the Southern Han Chinese. HLA-A*24:02, possibly HLA-A*30:01, are common risk factors for AED-MPE. The multiplicative risk potential between HLA-A*24:02 and HLA-B*38:02 suggests that patients with two risk alleles are at greater risk than those with one risk allele. Inclusion of these HLA alleles in pre-treatment screening would help estimating the risk of AED-MPE.

## Introduction

Aromatic antiepileptic drugs (AEDs), including carbamazepine (CBZ), lamotrigine (LTG), and oxcarbazepine (OXC), are commonly prescribed in clinical practice. However, these medications are also the most common causes of cutaneous adverse drug reactions (cADRs) (Arif et al., 2007; Mockenhaupt et al., 2008). The cADRs range from mild maculopapular exanthema (MPE), with increasing severity, to hypersensitivity syndrome (HSS), Stevens-Johnson syndrome (SJS), and toxic epidermal necrolysis (TEN) (Toledano and Gil-Nagel, 2008). MPE is relatively mild but generally requires medication discontinuation, disrupting AED treatment (Arif et al., 2007; Mockenhaupt et al., 2008). The overall incidence of MPE induced by AEDs (AED-MPE) is estimated to be 2.8%, and is higher at 3.7, 4.8, and 9% with CBZ, LTG, and OXC (Arif et al., 2007; Wang et al., 2012), respectively. CBZ, LTG, and OXC also have a 20–30% chance of cross-reactivity in MPE with other AEDs (Hirsch et al., 2008). MPE may evolve to severe SJS/TEN, which has high mortality of up to 30% (Roujeau, 1994). Several human leukocyte antigen (HLA) alleles have been demonstrated to be risk factors for CBZ-induced SJS/TEN, including HLA-B*15:02 in the Han Chinese and Southeast Asian populations (Chung et al., 2004; Tassaneeyakul et al., 2010; Shi et al., 2017), and HLA-A*31:01 in Japanese and European populations (McCormack et al., 2011; Ozeki et al., 2011), while HLA-A*24:02 is a common risk factor for AEDs-SJS/TEN (Shi et al., 2017). These findings have resulted in breakthroughs in the prevention of CBZ-induced SJS/TEN (Chen et al., 2011). Previously, several studies demonstrated that HLA alleles are possibly associated with AED-MPE, such as HLA-A*31:01 with CBZ-induced MPE (McCormack et al., 2011) and HLA-A*24:02 with LTG-induced MPE (Moon et al., 2015). However, the genetic HLA risk alleles of AED-MPE were largely undetermined.

To examine the HLA risk alleles in AED-MPE, we performed a multicenter case-control study with a cohort of 267 MPE cases from southern China. Significant findings were validated by meta-analyses of data from independent studies in other populations and computer simulation on protein-drug interactions^1^.

## Materials and Methods

### Recruitment of Cases and Drug-Tolerant Controls

The diagnoses of MPE were on the basis of the clinical morphology of the skin damage, which is characterized by cutaneous fine pink macules and papules, and lesions without mucosal or systemic involvement. A dermatologist confirmed the diagnosis. Cases with AED-MPE were determined according to the Naranjo algorithm (Naranjo. et al., 1981) and were recruited from six hospitals in three provinces in southern China, including Guangdong, Hunan, and Sichuan, between January 2007 and June 2019. These patients developed MPE within 8 weeks after receiving an aromatic AED. As a part of follow-up after AEDs were prescribed, the patients came to hospital when MPE were developed, and no other causes for the MPE were found. No cases were taking other medicines associated with cADRs such as abacavir and allopurinol. The tolerant controls were patients with epilepsy who received aromatic AEDs for at least 3 months without evidence of cutaneous adverse reactions. The controls were matched cases of Han Chinese from the same region who took the same AED, with a matching ratio of at least 1:1 (controls: cases). All individuals enrolled were unrelated ethnic southern Han Chinese. None of the four biological grandparents were from other races.

### Standard Protocol Approvals, Registrations, and Patient Consents

This study adhered to the guidelines of the International Committee of Medical Journal Editors with regard to patient consent for research or participation and received approvals from local ethics committees of the participating hospitals.

### HLA High-Resolution Genotyping

Genomic DNA was obtained from peripheral blood using a QIAamp blood mini kit (Qiagen, Hilden, Germany). High-resolution sequence-based HLA-A, HLA-B, HLA-C, and HLA-DRB1 typing was performed by Shanghai Tissuebank Biotechnology (Shanghai, China), as described previously (Shi et al., 2017).

### Meta-Analysis

To validate the potential risk alleles identified from the present cohort, we performed meta-analyses on data from other populations. We conducted a thorough biomedical literature search using PubMed, Embase, and the Cochrane Library. The following terms were used in our searches: “antiepileptic drugs” or “AEDs”, “HLA”, or “human leukocyte antigen”, “cutaneous adverse drug reactions” or “cADRs”, and “maculopapular eruption” or “MPE”. The last search was conducted on December 31, 2020.

Criteria for the selection of studies were: 1) the report was a case-control study on association between HLA and AED-cADRs; 2) the genotyping method and ethnicity were provided; 3) the carrier rate of HLA-A*24:02, HLA-A*30:01, HLA-B*35:01, HLA-B*38:02, and HLA-DRB1*04:06 in the cases and the tolerant controls was reported; 4) the most recent publication with the largest samples was selected when duplicate publications were identified. Exclusion criteria were: 1) case reports or case series, review articles, and basic genetic research; 2) repeat studies; 3) non-human studies.

Data managements and analyses were performed using RevMan 5.0.24 software (Cochrane Collaboration, Copenhagen, Denmark). A *p*-value of less than 0.1 and *I* ^2^-value of higher than 50% were defined study heterogeneity, under which the association was assessed using the random model, or not using the fixed model.

### Computer Simulation on Protein-Drug Docking

HLA molecules play a role in the development of cADRs potentially via binding with drugs (Illing et al., 2012; Norcross et al., 2012; Ostrov et al., 2012). Hence, the binding affinity between possible risk HLA molecules and AEDs were performed by *in silico* analysis. The three-dimensional (3D) structure of CBZ, LTG, OXC and its R enantiomer of monohydroxy derivative (R-MHD) and S-MHD, was downloaded from the PubChem database (https://pubchem.ncbi.nlm.nih.gov). The experimentally determined 3D protein structures included HLA-A*11:01, HLA-A*24:02, HLA-DRB1*01:01, and HLA-DRB1*04:01 (Protein Data Bank ID 6ID4.pdb (Gu et al., 2019), 2BCK.pdb (Cole et al., 2006), 3PDO.pdb (Gunther et al., 2010), and 4MCY.pdb (Scally et al., 2013), respectively). The structures of other HLA molecules were predicted by I-TASSER (Yang et al., 2015) based on the templates from the PDB database (https://www.rcsb.org/). A homology model of HLA-A molecule and a heterodimer model of HLA-DR complex (consists of HLA-DRA1 and HLA-DRB1 molecules) were used for docking by Rosseta docking_local_refine protocol (Lyskov and Gray, 2008). PyMOL align command was used for alignment. To set the same docking parameters, we aligned the 3D structural files of HLA-A molecules with HLA-A*24:02 as reference, and the files of HLA-DRB1 molecules with DRB1*04:01 as reference. We set the binding pocket of HLA-A in the condition of box center point at 25.453 Å (Å, *x*-axis), 20.524 Å (*y*-axis), and 13.601 Å (*z*-axis) with the box size of x = 16 Å, y = 30 Å, and z = 30 Å. The binding pocket of HLA-DRB1 was 43.798 Å (*x*-axis), 19.478 Å (*y*-axis), and −2.022 Å (*z*-axis) with a box size of x = 16 Å, y = 24 Å, and z = 23 Å. The search parameter exhaustiveness was set to 15. The Autodock Vina was used to construct the binding model and calculate the binding affinity between the drug and the target (Trott and Olson, 2010). The binding poses between drugs and HLA targets were shown by PyMOL 1.7.

### Statistical Analysis

Statistical analysis was performed using SPSS version 19.0 (SPSS Inc., Chicago, IL, United States). A 2-by-2 χ^2^ test was used to compare the allele carrier rates between groups. An independent *t*-test was performed to compare the sex ratio, mean age, and initial dosages between groups. *p*-values less than 0.05 (two-sided) were considered statistically significant. To achieve sufficient power, the corrected *p* values (*p*_c_) were estimated by using Bonferroni’s correction for multiple comparisons (*n* = 24 for HLA-A, 51 for HLA-B, 25 for HLA-C, and 26 for HLA-DRB1 in the CBZ-MPE group; *n* = 19 for HLA-A, 46 for HLA-B, 23 for HLA-C, and 26 for HLA-DRB1 in the LTG-MPE group; *n* = 16 for HLA-A, 37 for HLA-B, 18 for HLA-C, and 19 for HLA-DRB1 in the OXC-MPE group; *n* = 24 for HLA-A, 51 for HLA-B, 27 for HLA-C, and 26 for HLA-DRB1 in the pooled group). Logistic regression was used to analyze the possible interaction between two risk alleles.

## Results

### Characteristics of Cases with MPE and Tolerant Controls

A total of 267 cases with aromatic AED-MPE (146 CBZ-induced MPE, 67 LTG-induced MPE, and 54 OXC-induced MPE) and 387 tolerant controls (180 exposed to CBZ, 102 to LTG, and 133 to OXC, overlapped in some cases due to use of more than one AED in the same individual) were included. All cases were taking aromatic AEDs for treatment of epilepsy. Their clinical characteristics are summarized in Table 1. There was no significant difference between the cases and tolerant controls in age and sex ratio. The CBZ and LTG controls showed significantly higher exposure doses than the cases (*p* = 1.22 × 10^−9^ and *p* = 2.87 × 10^−5^, respectively). No significant difference in OXC dose was found between cases with OXC-induced MPE and OXC tolerant controls. The median latency to MPE after CBZ, LTG, and OXC administration was 11.59 days (range: 1–60), 13.51 days (range: 1–60), and 12 days (range: 1–40), respectively. The majority of cases (132/141, 93.6%) developed MPE within 30 days after drug treatment.

**TABLE 1:** Demographic variables and clinical features of AEDs-induced MPE and Controls.

	MPE (*n* = 267)	Controls (*n* = 387)	*p* value
Sex, *n* (%)	–	–	–
Male	144 (53.93%)	211 (54.52%)	0.99
Female	123 (46.07%)	176 (45.48%)	0.98
Age (years), mean (range)	24.81 ± 17.77 (1–91)	26.79 ± 17.13 (1–90)	0.15
Major comorbidities^a^, *n* (%)	0 (0%)	14 (3.6%)	0.002
CBZ exposure	MPE (*n* = 146)	Controls (*n* = 180)	
Dosage (mg/day), mean (range)	347.86 ± 174.13 (50–800, *n* = 70^c^)	540.17 ± 289.41 (50–1600, *n* = 175^c^)	1.22 × 10^−9^
Latency to MPE (days)^b^	11.59 ± 11.15 (1–60, *n* = 66^c^)	NA	
Concurrent aromatic AEDs, *n* (%)	1 (0.6%)	70 (38.89%)	9.52 × 10^−17^
Concurrent nonaromatic AEDs, *n* (%)	6 (4.11%)	81 (45%)	1.04 × 10^−16^
LTG exposure	MPE (*n* = 67)	Controls (*n* = 102)	
Dosage (mg/day), mean (range)	60.39 ± 62.43 (3.125–300, *n* = 43^c^)	131.66 ± 99.99 (25–1000, *n* = 101^c^)	2.87 × 10^−5^
Latency to MPE (days)^b^	13.51 ± 12.13 (1–60, *n* = 43^c^)	NA	
Concurrent aromatic AEDs, *n* (%)	3 (4.48%)	41 (40.20%)	2.27 × 10^−7^
Concurrent nonaromatic AEDs, *n* (%)	11 (16.42%)	65 (63.73%)	1.47 × 10^−9^
OXC exposure	MPE (*n* = 54)	Controls (*n* = 133)	
Dosage (mg/day), mean (range)	481.71 ± 223.59 (60–900, *n* = 38^c^)	501.90 ± 263.72 (25–1200, *n* = 79^c^)	0.77
Latency to MPE (days)^b^	12.00 ± 10.30 (1–40, *n* = 28^c^)	NA	
Concurrent aromatic AEDs, *n* (%)	1 (1.85%)	31 (23.31%)	4.14 × 10^−4^
Concurrent nonaromatic AEDs, *n* (%)	6 (11.11%)	28 (21.05%)	0.11

AEDs, antiepileptic drugs; CBZ, carbamazepine; LTG, lamotrigine; MPE, maculopapular exanthema; NA, Not applicable; OXC, oxcarbazepine.

a The 14 patients in the control group were those after cerebral vascular stroke and still receiving treatment for stroke.

b The latency to MPE in nine cases was more than one month (32, 39, 60, 33, 32, 36, 60, 35 and 40 days, respectively).

c Data missing in several individuals.

As shown in Table 2, in CBZ-induced MPE, the carrier rate of HLA-B*38:02 was higher in the case group (18/145, 12.41%) than in the tolerant group (10/179, 5.59%; *p* = 0.03; OR: 2.4; 95% CI: 1.07–5.37). In LTG-induced MPE, no significant allele was found when compared to the tolerant-control group.

**TABLE 2:** Risk HLA alleles for CBZ-, LTG-, and OXC-induced MPE.

Allele	HLA genotype/total, *n*/*N* (%)		Cases vs. controls		Prevalence (%)	Sensitivity (%)	Specificity (%)	PPV (%)	NPV (%)	NNT
	MPE^a^	Controls^a^	*p* Value	OR (95%CI)						
CBZ					3.7					
B*38:02	18/145 (12.41)	10/179 (5.59)	0.03	2.40 (1.07–5.37)		12.41	94.41	7.86	96.56	218
A*24:02	44/140 (31.43)	37/177(20.90)	0.033	1.73 (1.04–2.88)		31.43	79.10	5.46	96.78	86
/B*38:02										
OXC					9.0					
DRB1*04:06	8/51 (15.69)	1/94 (1.06)	0.002^b^	17.30 (2.10–142.72)		15.69	98.94	59.41	92.23	71
Pooled					2.8					
A*24:02	56/253 (22.13)	48/308 (15.58)	0.047	1.54 (1.00–2.36)		22.13	84.42	3.93	97.41	162
A*30:01	14/253 (5.53)	7/308 (2.27)	0.043	2.52 (1.00–6.34)		5.53	97.73	6.56	97.29	646
B*35:01	11/261 (4.21)	4/344 (1.16)	0.02	3.74 (1.18–11.88)		4.21	98.84	9.47	97.28	848
B*38:02	28/261 (10.73)	20/344 (5.81)	0.03	1.94 (1.07–3.54)		10.73	94.19	5.05	97.34	333
DRB1*04:06	17/259 (6.56)	9/343 (2.62)	0.02	2.61 (1.14–5.95)		6.56	97.38	6.73	97.31	544

CBZ, carbamazepine; CI, confidence interval; LTG, lamotrigine; MPE, maculopapular exanthema; NPV, negative predictive value; NNT, number need to test; OR, odds ratio; OXC, oxcarbazepine; PPV, positive predictive value.

a Several individuals were not subjected to HLA genotyping because of insufficient DNA. Due to 25 individuals were tolerant to CBZ and OXC, and 3 to LTG and OXC, we calculated these individuals by once when pooled analysis.

b Significant after Bonferroni’s correction, *p*_c_ = 0.038, *n* = 19 for HLA-DRB1*04:06 correction in OXC-induced MPE.

In OXC-induced MPE, the carrier rate of HLA-DRB1*04:06 was significantly higher in the case group than in the tolerant group (8/51, 15.69% *vs*. 1/94, 1.06%; *p* = 0.002; OR: 17.30; 95% CI: 2.10–142.72). After Bonferroni’s correction, the significant difference remained (*p*_*c*_ = 0.038). Among the patients taking OXC, 8 (88.9%) of nine patients with HLA-DRB1*04:06 developed OXC-induced MPE. HLA-DRB1*04:06 has a sensitivity of 15.69% and a specificity of 98.94% for OXC-induced MPE.

Considering the shared aromatic ring, we performed a pooled analysis on the three antiepileptic drugs induced MPE (Table 2). HLA-B*38:02 and HLA-DRB1*04:06 remained to be significantly associated with AED-MPE (*p* = 0.03 and *p* = 0.02, respectively). In addition, HLA-A*24:02, HLA-A*30:01, and HLA-B*35:01 were demonstrated to be weakly associated alleles. HLA-A*24:02 was presented in 22.13% (56/253) of cases and 15.58% (48/308) of tolerant controls (*p* = 0.047); HLA-A*30:01 was present in 5.53% (14/253) of cases and 2.27% (7/308) of tolerant controls (*p* = 0.043); and HLA-B*35:01 was present in 4.21% (11/261) of cases and 1.16% (4/344) of tolerant controls (*p* = 0.020).

Based on the prevalence of MPE in previous reports (estimated to be 3.7, 9, and 2.8% for CBZ, OXC, and AEDs, respectively, (Arif et al., 2007; Wang et al., 2012) the positive prediction values (PPVs) for the individual tests ranged from 3.93 to 59.41%, and the negative prediction values (NPVs) were higher than 92.2%. The number need to test (NNT) using HLA-B*38:02 to prevent one case with CBZ-induced MPE was 218, using HLA-DRB1*04:06 to prevent one case with OXC-induced MPE was 71, using HLA-A*24:02, A*30:01, B*35:01, and B*38:02 to prevent one case with AED-induced MPE was 162, 646, 848, and 333, respectively, (Table 2).

HLA-B*15:02 is a strong and specific risk factor for CBZ-induced SJS/TEN (Chung et al., 2004; Tassaneeyakul et al., 2010; Shi et al., 2017), but we did not find any significant association between HLA-B*15:02 and CBZ-induced MPE or AED-MPE in the present study (Supplementary Table S2).

HLA-A*31:01 was previously reported to be associated with CBZ-induced cADRs in the European population (McCormack et al., 2011). Our previous studies did not find such association in CBZ-induced SJS (Shi et al., 2017). In the present study, whenever using separate or pooled analysis, HLA-A*31:01 did not show significant association with MPE (Supplementary Table S2). The difference may be due to the HLA-A*31:01 associated with AED-cADRs having ethnic specificity. Conversely, the carrier rate of HLA-B*40:01 and HLA-C*12:03 were lower in the CBZ-induced MPE group than in the tolerant group (*p* = 1.91 × 10^−4^, OR: 0.37; 95% CI: 0.21–0.62; and *p* = 0.001, OR: 0.13; 95% CI: 0.03–0.55, respectively, Supplementary Table S2), suggesting negative correlations.

### Interaction Between Any Two HLA Alleles Located in Different Locus

The five potentially associated alleles are located in the HLA-A, HLA-B, or HLA-DRB1 separately and do not cluster as a haplotype (http://www.allelefrequencies.net/). We did not find any cluster from the data of this cohort, either (Supplementary Table S1). We then analyzed the potential interaction between any two alleles at different locus using logistic regression. In CBZ-induced MPE, the risk in patients who were positive for both HLA-A*24:02 and HLA-B*38:02 were significantly higher than in those who were positive for either HLA-A*24:02 or HLA-B*38:02 alone. A potential interaction between HLA-A*24:02 and HLA-B*38:02 was suggested (OR 7.29, Table 3). When combining the HLA-A*24:02 and HLA-B*38:02 into a single risk group (i.e., carriers of HLA-A*24:02 and/or HLA-B*38:02), the sensitivity increased to 31.43% for CBZ-induced MPE, with a specificity of 79.10%. The PPV and NPV for the individual combined HLA-A*24:02 and HLA-B*38:02 tests were 5.46 and 96.78%, and the NNT to prevent one case was 86 (Table 2). We did not find any other potential multiplicative interactions between the risk HLA alleles for the antiepileptic drugs-induced MPE.

**TABLE 3:** Excess risk from interaction between HLA-A*24:02 and HLA-B*38:02 alleles in CBZ-Induced MPE (logistic regression).

HLA-A*24:02	HLA-B*38:02	MPE, *n*	Controls, *n*	OR
+	+	5	1	7.29
+	−	26	27	1.40
−	+	13	9	2.11
−	−	96	140	1.0

CBZ, carbamazepine; HLA, human leukocyte antigen; MPE, maculopapular exanthema; OR, odds ratio.With the multiplicative model, the expected joint odds ratio was 2.95 (1.40 × 2.11) and the odds ratio of the multiplicative interaction was 2.47 (7.29/2.95). Under an additive model, the expected joint odds ratio was 2.51 (1.40 + 2.11–1) and the excess risk from interaction was 4.78 (7.29–2.51).

### Meta-Analysis

**FIGURE 1:**
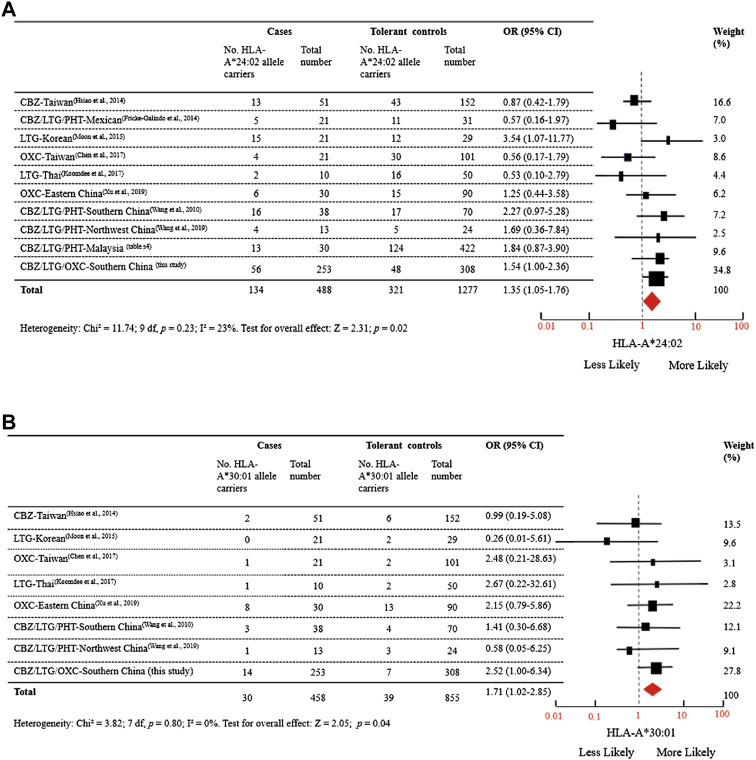
Distribution of HLA-A*24:02 and HLA-A*30:01 Alleles in MPE Induced by Aromatic Antiepileptic Drugs. **(A)** Data were from 10 studies relevant to the association between HLA-A*24:02 and AED-MPE, comprising of 488 cases with AED-MPE and 1277 AED-tolerant controls from China, Korea, Malaysia, Mexican, and Thai. **(B)** Data were from 8 studies relevant to the association between HLA-A*30:01 and AED-MPE, comprising of 458 cases with AED-MPE and 855 AED-tolerant controls from China, Korean, and Thai. *I* ^2^ represent measures of heterogeneity. Study weighting (indicated by different sizes of squares) refers to the proportion of participants who were recruited from each study. The horizontal lines represent 95% confidence intervals (CIs). Diamonds indicate pooled odds ratios. CBZ, carbamazepine; LTG, lamotrigine; MPE, maculopapular exanthema; OR, odds ratio, OXC, oxcarbazepine; PHT, phenytoin.

To test the significant association between these five alleles and AED-MPE, we employed meta-analysis of data from different studies and the data of the present study. A total of 152 published studies, and two unpublished data provided by the corresponding author (Supplementary Tables S3, S4) were collected. After exclusion, ten studies relevant to the association between HLA-A*24:02 and AED-MPE met the inclusion criteria, comprising of 488 cases with AED-MPE and 1277 AED-tolerant controls (Wang et al., 2010; Fricke-Galindo et al., 2014; Hsiao et al., 2014; Moon et al., 2015; Chen et al., 2017; Koomdee et al., 2017; Wang et al., 2019; Xu et al., 2019; Supplementary Table S4). The association between HLA-A*24:02 and AED-MPE was verified across different populations of China, Korea, Malaysia, Mexican and Thai (134/488 vs. 321/1277, *p* = 0.02, Figure 1A). HLA-A*30:01 was verified to be associated with AED-MPE based on data from eight studies including 458 cases and 855 tolerant controls (30/458 vs. 39/855, *p* = 0.04, Figure 1B). The other three alleles did not show significant association with AED-MPE (Supplementary Figure S1), but HLA-B*38:02 showed a tendency toward significant difference (33/357 vs. 37/644, *p* = 0.06).

### Computer Simulation on Binding of HLA Molecules to AEDs

The genotyping-comparison and meta-analysis indicated that HLA-A*24:02 and HLA-A*30:01 were risk alleles for AEDs-MPE and HLA-DRB1*04:06 was risk factor for OXC-induced MPE. We then investigated the docking situation between the risk molecules and antiepileptic drugs by computer simulation.

**FIGURE 2:**
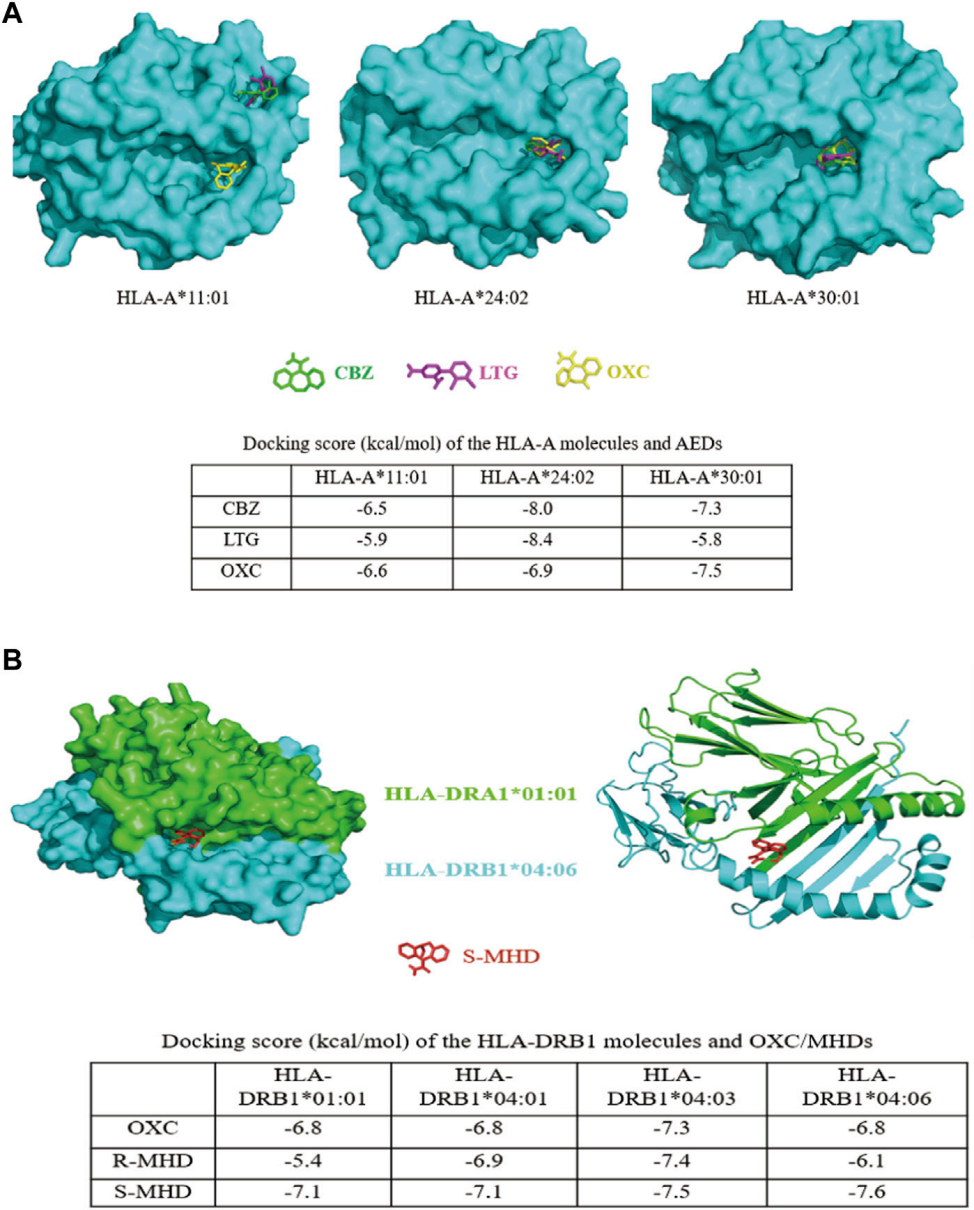
Three-dimensional models of interactions between HLA molecules and AEDs. **(A)** *In silico* modeling of interactions between the homology HLA-A molecules and AEDs. CBZ, LTG, and OXC were docked into the groove region of HLA-A*24:02 and HLA-A*30:01. For HLA-A*11:01, only OXC was docked into the groove region, while CBZ and LTG were docked into a region near the groove. The binding scores of HLA-A*24:02 with CBZ, LTG, and OXC, and HLA-A*30:01 with CBZ and OXC, were much higher than that of HLA-A*11:01 with the corresponding drugs (shown in the table). **(B)** *In silico* modeling of the interaction between the heterodimer of HLA-DRA1*01:01 and HLA-DRB1*04:06 and S-MHD. Similar to the model, OXC, R-MHD, and S-MHD were identically docked into the groove region of the HLA-DR molecules. The binding affinity of HLA-DRB1*04:06 and S-MHD (−7.6 kcal/mol) was the highest among 12 protein-drug interactions. HLA-DRB1*04:03 showed generally higher binding affinities with OXC, R-MHD, and S-MHD. All dock scores were listed in the table. CBZ, carbamazepine; LTG, lamotrigine; OXC, oxcarbazepine; R-MHD, R enantiomer of monohydroxy derivative; S-MHD, S enantiomer of monohydroxy derivative.

HLA-A*11:01 is the most frequent allele in general population but not a risk allele for AED-MPE. The protein structure of HLA-A*11:01 has been identified experimentally (Gu et al., 2019). We then analyzed the docking model of HLA-A*24:02 and HLA-A*30:01 with a comparison to HLA-A*11:01. For HLA-A*24:02 and HLA-A*30:01, the three antiepileptic drugs were docked well into the groove region. For HLA-A*11:01, only OXC was docked into the groove region, while CBZ and LTG were docked into a region near the groove region (Figure 2A). HLA-A*24:02 have generally higher binding affinity scores with CBZ, LTG, and OXC (−8.0, −8.4, and −6.9 kal/mol, respectively). HLA-A*30:01 also showed relatively higher binding affinity with CBZ and OXC (−7.3 and −7.5 kal/mol, respectively).

For HLA-DR, the protein structure of HLA-DRB1*01:01 and HLA-DRB1*04:01 have been identified experimentally (Gunther et al., 2010; Scally et al., 2013) and are risk-free molecules. Previously, HLA-DRB1*04:03 was showed to be a risk molecule for OXC-induced MPE in Korean (Moon et al., 2016). We therefore compared the docking models of HLA-DRB1*01:01, HLA-DRB1*04:01, HLA-DRB1*04:03, and HLA-DRB1*04:06. Considering OXC is rapidly metabolized to monohydroxylated derivatives (MHD) after oral administration in human (Flesch, 2004), we performed computer simulation on the docking of OXC and its metabolites R-MHD and S-MHD with the four heterodimer HLA-DR molecules. It was demonstrated that OXC, R-MHD, and S-MHD were identically docked into the groove region of the four molecules (Figure 2B and Supplementary Figure S2 (3-D protein-drug modeling figures provided, Supplementary Figure S2 is available on https://figshare.com/s/dae756a6f226a668db28), the docking model of S-MHD and HLA-DRB1*04:06 as a representative). HLA-DRB1*04:03 molecule showed generally higher binding affinities with OXC, R-MHD, and S-MHD (−7.3, −7.4, and −7.5 kcal/mol, respectively). However, HLA-DRB1*04:06 revealed the highest affinity with S-MHD (−7.6 kcal/mol), which is the main compound after oral taking of OXC in human (Flesch, 2004).

## Discussion

In the present study, we performed a multicenter case-control study and identified HLA-DRB1*04:06 as a specific risk factor for OXC-induced MPE and potentially HLA-B*38:02 as a risk allele for CBZ-induced MPE in Southern Han Chinese. Further studies suggest a multiplicative interaction between HLA-A*24:02 and HLA-B*38:02 in CBZ-induced MPE and that patients with two risk alleles are at greater risk than those with one risk allele alone. Meta-analysis on data from different populations revealed that HLA-A*24:02 and HLA-A*30:01 alleles were possibly common risk factors for AED-MPE. These genetic associations were supported by evidence from computer simulation on interactions between HLA molecules and AEDs.

OXC, a 10-keto analog of CBZ with less frequent and severe cADRs than CBZ (He et al., 2012; Chen et al., 2017), is commonly used as an alternative to CBZ in clinics. However, several studies reported that the incidence of OXC-induced cADRs was as high as 5–9% (Alvestad et al., 2007; Hirsch et al., 2008; Wang et al., 2012), resulting in drug discontinuation in almost all cases (Shorvon, 2000; Kalis and Huff, 2001; Wang et al., 2012). To date, the risk factors for OXC-induced MPE are largely unknown. In this multicenter case-control study in southern Han Chinese, we found that HLA-DRB1*04:06 was associated with OXC-induced MPE with high OR value (17.30), which remained significant difference after Bonferroni’s correction. Among the 145 epilepsy patients treated with OXC, eight (88.89%) of nine patients with HLA-DRB1*04:06 developed MPE. The high specificity (98.94%) and positive predictive value (88.89%) suggest potential implication of HLA-DRB1*04:06 detection in clinical practice. Therefore, the patients who were positive for HLA-DRB1*04:06 should be carefully looked when OXC was prescribed. However, the sensitivity of HLA-DRB1*04:06 for OXC-induced MPE is 15.96%, which may be due to the relatively low frequency (2.1%) of HLA-DRB1*04:06 in the Southern Han Chinese. HLA-DRB1*04:06 presents at frequencies of 2.1–3.5% in Chinese and 0–6.8% in other populations (www.allelefrequencies.net/default.asp). Given that the incidence of OXC-induced MPE is of up to 9% (Wang et al., 2012), the PPV and NPV of this allele are 59.41 and 92.25%, respectively. The NNT using HLA-DRB1*04:06 to prevent one case with OXC-induced MPE is 71.

AED-MPE is mainly mediated by CD4^+^ T cells recognized by HLA class II molecules (Pichler, 2002), which include the molecules encoded by HLA-DR, HLA-DQ, or HLA-DP alleles. HLA-DRB1*04:06 was the first risk HLA-DR molecule identified for OXC-induced MPE in the Southern Han Chinese. Similarly, HLA-DRB1*04:03 has been identified as a potential risk factor for OXC-induced MPE in the Korean population with a specificity of 98.57% and a sensitivity of 17.50% (Moon et al., 2016). HLA-DRB1*04:03 is also a less frequent allele in the Korean population (2.96%, www.allelefrequencies.net/default.asp). Generally, TCRs monitor the universe of antigens to which an individual is exposed by surveying the ligands (or peptides) presented on an antigen-presenting cell membrane. Different HLA molecules have different binding specificities, resulting in a specific profile of presented peptides. When T cells encounter an unknown peptide, an immune response is triggered. In case of cutaneous adverse drug reactions (cADRs), HLA molecule, drug, peptide, and T cell receptor (TCRs) are potentially involved. Previously, three models have been proposed to explain the underlying mechanism of immune-mediated cADRs: 1) a peptide, which is uniquely presented by the HLA allele, is modified by the drug; 2) the HLA molecule itself is modified by the drug in a region and then exposed to the TCR; 3) the binding specificity of the HLA molecule with peptide is altered by the presence of drug. In the latter two models, the drug needs to bind HLA molecules. Several studies have revealed that abacavir could bind inside the peptide-binding groove of HLA-B*57:01 to alter the repertoire of endogenous peptides, resulted in affecting TCR interface to trigger HLA-associated drug hypersensitivity, provided experimental evidence supporting the third model (Illing et al., 2012; Norcross et al., 2012; Ostrov et al., 2012). Based on these previous studies, we analyzed the binding of culprit AEDs with HLA molecules. Among of 12 drug-protein interactions, the docking score of S-MHD binding with HLA-DRB1*04:06 was the highest (Figure 2B). After oral administration of OXC in human, only 2% of the total doses is present as OXC, approximately 70% as MHDs, and the others are rapidly eliminated. Of the MHDs, the S-MHD over R-MHD ratio is 4:1 (Flesch, 2004). Therefore, the strong interaction between HLA-DRB1*04:06 and S-MHD potentially explained HLA-DRB1*04:06 as a risk allele in OXC-induced MPE in the Southern Han Chinese. However, it is uncertain whether AEDs binds with the risk HLA allele to change the shape and chemistry of the antigen-binding cleft, thereby altering the repertoire of endogenous peptides that can bind the allele. In addition, the dock scores between HLA-DRB1*04:03 and OXC, R-MHD, and S-MHD were relatively high, potentially explaining the association between HLA-DRB1*04:03 and OXC-MPE in the Korean population. The mechanisms underlying the tiny difference of risk allele between the two populations are unknown, but ethnic specificity may be one of explanations (Lee et al., 2010), such as endogenous peptides and T cell receptors (Geursen et al., 1993; Ko et al., 2011).

We previously reported that HLA-A*24:02 was a common risk factor for AED-SJS/TEN with a tendency of risk for MPE (Shi et al., 2017). In this study, we compared a relatively large cohort comprising of CBZ, LTG, and OXC induced-MPE to tolerant controls (Table 2) and performed meta-analysis of the data from different populations (Figure 1A) to demonstrate that HLA-A*24:02 was a potentially common risk allele for AED-MPE. The binding scores of CBZ, LTG, and OXC with HLA-A*24:02 molecule were much higher than that of the risk-free HLA-A*11:01 molecule (Figure 2A), providing additional evidence of HLA-A*24:02 as a common risk for cADRs. Similarly, HLA-A*30:01 was suggested to be a possibly common risk factor for AED-MPE (Figure 1A and Figure 2A).

Previous studies indicated that some HLA alleles appeared to be a specific risk for AED-cADRs, such as HLA-B*15:02 for CBZ-induced SJS/TEN (Chung et al., 2004), whereas other HLA alleles showed common risk potential, such as HLA-A*24:02 for AED-SJS/TEN (Shi et al., 2017). In the present study, HLA-DRB1*04:06 was demonstrated as a specific risk factor for OXC-induced MPE, while HLA-A*24:02 and HLA-A*30:01 as potentially common risk factors for AED-MPE (Table 1). These findings may be explained by highly polymorphic HLA alleles and the highly heterogeneous binding affinities of the risk HLA molecules with AEDs.

HLA alleles are co-expressed on the cell surface. Our previous study demonstrated that patients with two risk alleles are at greater risk for developing AED-SJS/TEN than those with one risk allele (Shi et al., 2017). In the present study, we found the multiplicative risk potential between HLA-A*24:02 and HLA-B*38:02 (Table 3). The patients with the two alleles increased about 3–5 times the risk for CBZ-induced MPE when compared to the patients with one allele alone. In addition, the NNT using the two risk alleles to prevent one case with CBZ-induced MPE is decreased to 86 (Table 2). Therefore, attentions should be paid to the multiplicative risk potential of HLA alleles.

A recent study reported that HLA-B*15:02 and HLA-B*58:01 were associated with CBZ-induced MPE in the Thai population (Sukasem et al., 2018). Our previous study found that HLA-B*15:02 was associated with CBZ-induced SJS/TEN (Shi et al., 2017). In this study, we did not find its association with CBZ-induced MPE in the Han Chinese population. Another risk allele HLA-B*58:01 is worthy of verifying in more cases with CBZ-induced MPE. Similarly, HLA-A*31:01 was reported to be a risk factor for CBZ-induced cADRs in the European population (McCormack et al., 2011), we did not find such association in the Han Chinese population, in our previous study (Shi et al., 2017), as well as in the present study. The differences may be explained by the different ethnicity. According to the Clinical Pharmacogenetics Implementation Consortium (CPIC) guidelines, HLA-A*31:01 and HLA-B*15:02 should be tested before taking CBZ and CBZ/OXC in patients firstly prescribed, respectively, (Leckband et al., 2013; Phillips et al., 2018). From our findings, the efficacy of HLA-A*31:01 testing for the Han Chinese population should be evaluated further.

The present study had some limitations. We found several HLA alleles to be associated with AED-MPE, but their risk potential was not as strong as that of HLA alleles associated with AED-SJS/TEN (Chung et al., 2004; Shi et al., 2017). MPE is a milder phenotype of cADRs, which would generally be affected by other factors (He et al., 2012; McCormack et al., 2018) that we did not consider in this study. Further efforts are required to identify novel HLA or other genetic risk alleles of cADRs, including that for specific populations. We only analyzed the binding of culprit AEDs with HLA molecules, not considering the complicated neo-self peptides specifically in the presence of AEDs and the complexity of TCRs, which should be investigated further. In addition, HLA-B*40:01 and HLA-C*12:03 were found to be negatively associated with AED-MPE (Supplementary Table S2). However, the precise role of these alleles in the developing of cADRs remains unknown.

In summary, our data suggest that HLA-DRB1*04:06 is a highly specific risk factor for OXC-induced MPE in the Southern Han Chinese and that HLA-A*24:02, possibly HLA-A*30:01 are common risk factors for AED-MPE. Inclusion of these HLA alleles in pre-treatment screening would help estimate the risk of AED-MPE in individuals.

## Data Availability

The original contributions presented in the study are included in the article/Supplementary Material, further inquiries can be directed to the corresponding author.

## Footnotes

1 For the China Epilepsy Gene 1.0 Project: www.epg1.cn
