# Supplementary material for: HLA Risk Alleles in Aromatic Antiepileptic Drug-Induced Maculopapular Exanthema
Source: Front Pharmacol. 2021 May 26;12:671572. doi: 10.3389/fphar.2021.671572 (PMC8187898; doi:10.3389/fphar.2021.671572)
Supplement: Supplementary file 1 [file DataSheet2.PDF]

FIGURE S2 is available on https://figshare.com/s/dae756a6f226a668db28

FIGURE S2. Three-dimensional model of the interaction between the heterodimer of HLA-DRA1*01:01/HLA-DRB1*04:06 and S enantiomer of monohydroxy derivative of oxcarbazepine.
