# Supplementary material for: HLA Risk Alleles in Aromatic Antiepileptic Drug-Induced Maculopapular Exanthema
Source: Front Pharmacol. 2021 May 26;12:671572. doi: 10.3389/fphar.2021.671572 (PMC8187898; doi:10.3389/fphar.2021.671572)
Supplement: Supplementary file 2 [file DataSheet1.DOC]

**Supplementary material**

**This file includes:**

**Tables (S1, S2, S3, and S4).**

**Table S1** HLA genotypes in patients with CBZ-, LTG-, and OXC-induced MPE

| **Patient no.** | **Culprit drug** | **HLA-A genotype** | **HLA-B genotype** | **HLA-C genotype** | **HLA-DRB1 genotype** |
| --- | --- | --- | --- | --- | --- |
| 1 | CBZ | 11:01/33:03 | 15:12/58:01 | 03:02/03:04 | 03:01/15:02 |
| 2 | CBZ | None | 15:25/46:01 | None | None |
| 3 | CBZ | 24:02/24:02 | 40:01/54:01 | 01:02/07:02 | 04:05/14:05 |
| 4 | CBZ | 02:03/02:07 | 38:01/46:01 | 01:02/07:02 | 09:01/16:02 |
| 5 | CBZ | 11:01/11:02 | 15:27/13:01 | 03:04/04:01 | 04:06/12:02 |
| 6 | CBZ | 02:06/11:02 | 15:01/13:01 | 03:04/08:01 | 12:02/16:02 |
| 7 | CBZ | 02:03/26:01 | 15:02/15:19 | 03:03/08:01 | 12:02/12:02 |
| 8 | CBZ | 02:07/02:07 | 46:01/46:01 | 01:02/01:02 | 09:01/09:01 |
| 9 | CBZ | 02:03/24:02 | 13:01/44:03 | 03:04/12:02 | 15:01/15:01 |
| 10 | CBZ | 11:01/11:01 | 40:01/46:01 | 01:02/07:02 | 04:05/07:01 |
| 11 | CBZ | 02:07/11:01 | 15:01/46:01 | 01:02/04:01 | 14:05/14:05 |
| 12 | CBZ | 02:01/02:01 | 13:01/46:01 | 01:02/03:04 | 04:06/16:02 |
| 13 | CBZ | 02:07/33:03 | 46:01/58:01 | 01:02/03:02 | 03:01/09:01 |
| 14 | CBZ | 24:02/33:03 | 15:02/15:02 | 07:02/08:01 | 08:03/12:02 |
| 15 | CBZ | 11:01/33:03 | 13:01/27:02 | 03:04/14:03 | 13:02/15:01 |
| 16 | CBZ | 02:03/11:01 | None | 03:04/03:04 | 12:02/15:01 |
| 17 | CBZ | 24:02/24:02 | 15:02/40:01 | 04:01/07:02 | 04:05/04:06 |
| 18 | CBZ | 11:01/33:03 | 39:01/58:01 | 03:02/07:02 | 03:01/14:05 |
| 19 | CBZ | 02:07/11:01 | 46:01/46:01 | 01:02/01:03 | 09:01/09:01 |
| 20 | CBZ | 11:01/33:03 | 52:01/52:01 | 03:02/07:02 | 03:01/16:02 |
| 21 | CBZ | 02:07/11:01 | 15:01/40:01 | 01:02/07:02 | 08:03/09:01 |
| 22 | CBZ | 02:03/11:02 | 35:01/38:02 | 03:04/07:02 | 15:01/16:02 |
| 23 | CBZ | 02:01/33:03 | 40:01/58:01 | 03:02/15:02 | 11:01/12:02 |
| 24 | CBZ | 24:02/33:03 | 58:01/58:01 | 03:02/03:02 | 03:01/03:01 |
| 25 | CBZ | 11:01/11:01 | 15:02/13:01 | 03:04/08:01 | 09:01/12:02 |
| 26 | CBZ | 11:01/31:01 | 15:02/15:02 | 08:01/15:02 | 12:02/12:02 |
| 27 | CBZ | 02:06/02:06 | 15:25/38:02 | 04:03/07:02 | 08:03/09:01 |
| 28 | CBZ | 02:01/11:01 | 13:02/56:01 | 01:02/03:04 | 12:02/15:02 |
| 29 | CBZ | 11:01/11:01 | 15:02/40:02 | 03:04/08:01 | 04:03/12:02 |
| 30 | CBZ | 02:07/03:01 | 46:01/46:01 | 01:02/15:02 | 01:01/09:01 |
| 31 | CBZ | 02:06/24:02 | 15:02/46:01 | 01:02/0841 | 09:01/12:02 |
| 32 | CBZ | 24:02/24:02 | 39:01/48:01 | 07:02/15:02 | 12:02/15:01 |
| 33 | CBZ | 11:01/26:01 | 15:01/15:01 | 03:02/04:01 | 03:01/14:05 |
| 34 | CBZ | 11:01/24:02 | 15:01/15:01 | 04:01/14:02 | 04:06/11:01 |
| 35 | CBZ | 02:03/02:07 | 13:01/55:02 | 03:04/12:03 | 12:02/15:01 |
| 36 | CBZ | 11:01/11:01 | 15:02/1518 | 02:02/08:01 | 04:04/15:01 |
| 37 | CBZ | 11:01/31:01 | 15:01/56:04 | 01:02/03:03 | 14:54/15:02 |
| 38 | CBZ | 02:03/31:01 | 5901/5107 | 07:02/15:02 | 08:03/16:02 |
| 39 | CBZ | 02:03/24:02 | None | 03:04/08:01 | 12:01/12:02 |
| 40 | CBZ | None | 40:01/40:01 | 03:04/03:04 | 09:01/15:02 |
| 41 | CBZ | 11:02/33:03 | 13:01/55:02 | 01:02/03:04 | 13:12/16:02 |
| 42 | CBZ | 03:01/31:01 | 46:01/54:01 | 01:02/01:02 | 04:05/12:02 |
| 43 | CBZ | 01:01/11:01 | 15:02/1517 | 07:01/08:01 | 12:02/13:02 |
| 44 | CBZ | 02:07/24:02 | 46:01/51:01 | 01:02/14:02 | 11:01/14:54 |
| 45 | CBZ | None | 13:01/46:01 | 01:02/03:04 | 12:02/14:54 |
| 46 | CBZ | 24:02/74:02 | 46:01/51:01 | 01:02/14:02 | 09:01/14:04 |
| 47 | CBZ | 02:07/33:03 | 15:27/27:04 | 01:02/07:06 | 09:01/14:54 |
| 48 | CBZ | 02:07/24:02 | 13:01/52:01 | 03:04/12:02 | 13:02/15:01 |
| 49 | CBZ | None | 51:01/58:01 | 03:02/14:02 | 03:01/15:01 |
| 50 | CBZ | 30:01/30:01 | 13:02/13:02 | 06:02/06:02 | 07:01/07:01 |
| 51 | CBZ | 11:01/24:02 | 39:01/40:01 | 07:02/07:02 | 13:12/14:18 |
| 52 | CBZ | 11:01/02:07 | 15:02/46:01 | 01:02/08:01 | 09:01/09:01 |
| 53 | CBZ | 02:07/02:07 | 46:01/46:01 | 01:02/01:03 | 14:54/15:01 |
| 54 | CBZ | 02:01/33:03 | 13:01/38:02 | 03:04/07:02 | 08:03/09:01 |
| 55 | CBZ | 02:01/02:07 | 38:02/46:01 | 01:02/07:02 | 09:01/14:54 |
| 56 | CBZ | 33:03/33:03 | 58:01/58:01 | 03:02/03:02 | 03:01/11:01 |
| 57 | CBZ | 11:01/74:02 | 13:01/51:01 | 08:01/14:02 | 03:01/14:04 |
| 58 | CBZ | 11:01/11:01 | 40:01/40:01 | None | None |
| 59 | CBZ | 11:01/11:02 | 15:02/40:01 | 07:02/08:01 | 12:02/15:01 |
| 60 | CBZ | 02:03/11:01 | 13:01/13:01 | 03:04/03:04 | 12:02/16:02 |
| 61 | CBZ | 02:06/11:02 | 13:01/27:04 | 03:04/12:02 | 15:01/15:01 |
| 62 | CBZ | 24:02/26:01 | 67:01/67:01 | 07:02/07:02 | 16:02/16:02 |
| 63 | CBZ | 02:01/11:01 | 15:02/15:11 | 03:03/08:01 | 09:01/12:02 |
| 64 | CBZ | 02:01/24:20 | 13:01/40:01 | 03:02/07:02 | 12:02/15:01 |
| 65 | CBZ | 11:01/24:02 | 35:05/46:01 | 01:02/07:02 | 09:01/12:02 |
| 66 | CBZ | 11:01/24:02 | 13:01/40:01 | 03:04/07:02 | 15:01/16:02 |
| 67 | CBZ | 30:01/31:01 | 13:02/40:01 | 06:02/15:02 | 07:01/12:02 |
| 68 | CBZ | 02:06/02:07 | 35:01/46:01 | 01:02/03:03 | 08:03/15:01 |
| 69 | CBZ | 11:01/11:01 | 13:01/35:01 | 03:03/03:04 | 09:01/15:01 |
| 70 | CBZ | 11:01/11:01 | 15:01/40:06 | None | 11:01/12:01 |
| 71 | CBZ | 02:07/02:07 | 46:01/46:01 | 01:02/01:02 | 09:01/09:01 |
| 72 | CBZ | 02:01/11:01 | 15:25/39:01 | 04:03/07:02 | 09:01/16:02 |
| 73 | CBZ | 02:06/24:02 | 13:01/40:01 | 03:04/03:04 | 11:01/14:05 |
| 74 | CBZ | 11:01/11:01 | 13:01/35:01 | 03:03/03:04 | 09:01/15:01 |
| 75 | CBZ | 02:03/11:01 | 13:01/38:02 | 03:04/07:02 | 12:02/14:05 |
| 76 | CBZ | 02:03/24:02 | 15:02/46:01 | 01:02/08:01 | 12:02/12:02 |
| 77 | CBZ | 11:01/31:01 | 15:02/35:01 | 04:01/08:01 | 11:01/15:01 |
| 78 | CBZ | 02:07/11:01 | 46:01/46:01 | 01:02/01:02 | 09:01/09:01 |
| 79 | CBZ | 02:07/11:01 | 27:04/46:01 | 01:02/12:02 | 09:01/12:02 |
| 80 | CBZ | 02:07/11:02 | 46:01/46:01 | 01:02/01:02 | 09:01/13:12 |
| 81 | CBZ | 02:01/24:02 | 50:01/50:02 | 01:02/06:02 | 03:01/04:05 |
| 82 | CBZ | 02:03/02:07 | 38:02/46:01 | 01:02/07:02 | 09:01/16:02 |
| 83 | CBZ | 11:01/11:02 | 13:01/58:01 | 03:02/03:04 | 03:01/15:01 |
| 84 | CBZ | 02:03/02:07 | 38:02/46:01 | 01:02/07:02 | 08:03/16:02 |
| 85 | CBZ | 11:01/11:01 | 40:06/48:01 | 03:03/08:01 | 09:01/12:02 |
| 86 | CBZ | 02:07/26:01 | 40:02/46:01 | 01:02/03:03 | 09:01/11:01 |
| 87 | CBZ | 11:01/24:02 | 15:02/46:01 | 01:02/08:01 | None |
| 88 | CBZ | 24:02/30:01 | 13:01/13:02 | 03:04/06:02 | 07:01/12:02 |
| 89 | CBZ | 02:07/31:01 | 40:01/51:01 | 07:02/14:02 | 04:04/14:54 |
| 90 | CBZ | 11:01/33:03 | 15:02/51:02 | 03:02/08:01 | 04:03/12:02 |
| 91 | CBZ | 11:01/11:01 | 15:01/52:01 | 01:02/12:02 | 12:01/15:01 |
| 92 | CBZ | 02:01/02:06 | 38:02/40:01 | 07:02/15:02 | 11:01/16:02 |
| 93 | CBZ | 11:01/24:02 | 13:01/13:01 | 03:04/03:04 | 08:03/15:01 |
| 94 | CBZ | 02:03/24:03 | 38:02/40:02 | 03:04/07:02 | 15:02/16:02 |
| 95 | CBZ | 11:01/30:01 | 13:01/13:02 | 03:04/06:02 | 07:01/15:01 |
| 96 | CBZ | 11:01/33:03 | 15:02/58:01 | 03:02/08:01 | 03:01/15:01 |
| 97 | CBZ | 02:07/11:01 | 15:01/46:01 | 01:02/04:01 | 04:06/16:02 |
| 98 | CBZ | 02:03/24:02 | 38:02/54:01 | 01:02/07:02 | 08:03/14:05 |
| 99 | CBZ | 11:01/11:01 | 15:02/51:01 | 08:01/14:02 | 15:01/15:02 |
| 100 | CBZ | 11:01/30:01 | 13:02/40:02 | 06:02/07:02 | 07:01/12:02 |
| 101 | CBZ | 02:07/11:01 | 40:01/46:01 | 01:02/07:02 | 04:05/08:03 |
| 102 | CBZ | 02:03/24:02 | 38:02/46:01 | 01:02/07:02 | 09:01/15:02 |
| 103 | CBZ | 11:01/33:03 | 13:01/58:01 | 03:02/03:04 | 12:02/15:01 |
| 104 | CBZ | 11:01/31:01 | 15:02/46:01 | 01:02/08:01 | 04:04/12:02 |
| 105 | CBZ | 02:07/03:01 | 46:01/51:01 | 01:02/15:02 | 01:01/09:01 |
| 106 | CBZ | 02:07/11:01 | 46:01/55:12 | 01:02/01:02 | 04:05/12:02 |
| 107 | CBZ | 11:01/24:02 | 15:01/38:02 | 03:03/08:01 | 15:01/15:02 |
| 108 | CBZ | 02:03/33:03 | 38:02/58:01 | 03:02/07:02 | 03:01/16:02 |
| 109 | CBZ | 02:07/30:01 | 13:02/46:01 | 01:02/06:02 | 07:01/09:01 |
| 110 | CBZ | 02:03/11:01 | 40:06/56:04 | 01:02/07:02 | 09:01/15:02 |
| 111 | CBZ | 11:01/11:01 | 38:02/40:01 | 03:04/07:02 | 04:03/12:02 |
| 112 | CBZ | 02:03/11:01 | 40:01/51:01 | 03:17/14:02 | 04:05/14:54 |
| 113 | CBZ | 11:01/11:01 | 40:01/46:01 | 01:02/07:02 | 08:03/09:01 |
| 114 | CBZ | 02:07/31:01 | 13:01/46:01 | 01:02/15:02 | 08:03/16:02 |
| 115 | CBZ | 11:01/68:01 | 08:01/55:02 | 01:02/07:02 | 03:01/04:05 |
| 116 | CBZ | 11:01/11:01 | 13:01/51:01 | 03:04/1504 | 12:02/12:02 |
| 117 | CBZ | 11:01/24:02 | 15:27/39:01 | 04:01/07:02 | 08:03/12:02 |
| 118 | CBZ | 02:07/11:01 | 13:01/18:02 | 03:04/07:04 | 12:02/12:02 |
| 119 | CBZ | 02:06/02:07 | 46:01/46:01 | 01:02/01:02 | 14:54/14:54 |
| 120 | CBZ | 11:01/11:01 | 15:01/39:05 | 04:01/07:02 | 12:02/16:02 |
| 121 | CBZ | 11:01/24:02 | 27:04/40:01 | 03:04/12:02 | 11:01/15:01 |
| 122 | CBZ | 0210/33:03 | 46:01/58:01 | 03:02/08:01 | 04:06/12:01 |
| 123 | CBZ | 24:02/31:01 | 15:02/39:01 | 07:02/08:01 | 12:02/14:18 |
| 124 | CBZ | 02:07/11:02 | 39:01/46:01 | 01:02/07:02 | 14:01/14:18 |
| 125 | CBZ | 02:07/24:02 | 38:02/46:01 | 01:02/07:02 | 09:01/16:02 |
| 126 | CBZ | 02:06/11:01 | 13:01/48:03 | 03:04/08:01 | 09:01/11:01 |
| 127 | CBZ | 02:07/11:01 | 46:01/55:02 | 01:02/07:02 | 08:03/14:54 |
| 128 | CBZ | 02:03/02:07 | 15:19/46:01 | 01:02/04:03 | 15:01/15:01 |
| 129 | CBZ | 26:01/32:01 | 40:02/52:01 | 01:02/12:02 | 13:02/15:02 |
| 130 | CBZ | 11:01/11:01 | 13:01/13:01 | 03:04/03:04 | 15:01/15:01 |
| 131 | CBZ | 11:01/33:03 | 35:01/58:01 | 03:02/15:02 | 03:01/08:03 |
| 132 | CBZ | 11:02/11:02 | 13:01/58:01 | 03:02/03:04 | 03:01/14:05 |
| 133 | CBZ | 02:03/02:07 | 38:02/46:01 | 01:02/07:02 | 07:01/16:02 |
| 134 | CBZ | 24:02/33:03 | 15:02/58:01 | 03:02/08:01 | 12:02/13:02 |
| 135 | CBZ | 02:01/11:01 | 27:04/39:01 | 07:02/08:01 | 08:03/15:01 |
| 136 | CBZ | 02:03/24:02 | 35:03/38:02 | 07:02/12:03 | 14:04/16:02 |
| 137 | CBZ | 02:01/30:01 | 07:02/13:02 | None | 07:01/15:01 |
| 138 | CBZ | 11:01/24:10 | 13:01/18:02 | 03:04/07:04 | 12:02/15:01 |
| 139 | CBZ | 02:01/30:01 | 13:02/40:01 | 03:04/06:02 | 04:06/07:01 |
| 140 | CBZ | 02:03/11:01 | 15:02/40:01 | 03:04/08:01 | 12:02/16:02 |
| 141 | CBZ | 02:07/02:07 | 46:01/55:12 | 01:02/01:02 | 04:05/09:01 |
| 142 | CBZ | 02:01/02:06 | 35:01/51:02 | 01:02/03:03 | 09:01/09:01 |
| 143 | CBZ | 11:01/24:02 | 40:01/40:01 | 03:04/07:02 | 12:02/15:01 |
| 144 | CBZ | 02:03/02:07 | 38:02/46:01 | 01:02/07:02 | 07:01/16:02 |
| 145 | CBZ | 02:03/11:01 | 13:01/51:02 | 03:04/14:02 | 12:02/15:01 |
| 146 | CBZ | 02:07/11:01 | 40:01/46:01 | 01:02/07:02 | 04:03/11:01 |
| 147 | LTG | 02:07/11:01 | 15:02/46:01 | 01:02/04:01 | 09:01/11:01 |
| 148 | LTG | 02:03/29:01 | 55:02/55:02 | 12:03/15:05 | 10:01/11:01 |
| 149 | LTG | 11:01/26:01 | 40:01/40:01 | None | None |
| 150 | LTG | 02:03/33:03 | 38:02/44:03 | 07:02/14:03 | 15:01/16:02 |
| 151 | LTG | 02:01/02:06 | 39:01/48:01 | 03:04/08:22 | 04:05/04:07 |
| 152 | LTG | None | 54:01/58:01 | 01:02/03:02 | 03:01/04:05 |
| 153 | LTG | 02:07/02:07 | 46:01/58:01 | 01:03/03:02 | 03:01/09:01 |
| 154 | LTG | 24:02/24:02 | 40:01/81:01 | 01:02/01:02 | 14:05/14:54 |
| 155 | LTG | 11:01/24:02 | 40:01/55:02 | 01:02/07:02 | 08:03/13:12 |
| 156 | LTG | 11:01/24:02 | 40:01/40:01 | 03:04/07:02 | 08:03/14:54 |
| 157 | LTG | 02:03/11:01 | 46:01/46:01 | 03:04/07:02 | 09:01/12:02 |
| 158 | LTG | 02:07/11:01 | 13:01/46:01 | 01:02/03:04 | 09:01/09:01 |
| 159 | LTG | 11:01/30:01 | 13:02/13:02 | 06:02/15:02 | 04:03/07:01 |
| 160 | LTG | 02:01/33:03 | 08:01/13:02 | 06:02/07:02 | 07:01/13:02 |
| 161 | LTG | 02:03/11:01 | 15:19/15:19 | 04:03/12:02 | 12:01/12:02 |
| 162 | LTG | 02:07/24:02 | 15:25/46:01 | 01:02/03:04 | 04:05/12:02 |
| 163 | LTG | None | 15:02/46:01 | 01:02/08:01 | 09:01/12:02 |
| 164 | LTG | None | None | 03:02/08:01 | 03:01/12:02 |
| 165 | LTG | 02:07/02:07 | 15:02/46:01 | 01:02/08:01 | 14:54/15:01 |
| 166 | LTG | 11:01/32:01 | 38:02/52:01 | 07:02/12:02 | 01:01/15:02 |
| 167 | LTG | 02:03/11:01 | 38:02/40:01 | 03:04/07:02 | 13:12/15:01 |
| 168 | LTG | None | 15:01/46:01 | 01:02/03:03 | 04:05/09:01 |
| 169 | LTG | 02:07/33:03 | 54:01/01:02 | 12:03/04:05 | 04:05/14:54 |
| 170 | LTG | 02:07/11:01 | 46:01/5601 | 01:02/01:03 | 09:01/14:54 |
| 171 | LTG | 30:01/30:01 | 13:02/13:02 | 06:02/06:02 | 07:01/07:01 |
| 172 | LTG | 02:01/11:01 | 1513/35:03 | 04:01/08:01 | 13:01/15:01 |
| 173 | LTG | 11:01/02:07 | 15:02/46:01 | 01:02/08:01 | 09:01/09:01 |
| 174 | LTG | 11:01/02:07 | 15:02/40:01 | 07:02/08:01 | 04:05/12:02 |
| 175 | LTG | 02:01/33:03 | 13:01/38:02 | 03:04/07:02 | 08:03/09:01 |
| 176 | LTG | 11:01/11:01 | 15:01/15:02 | 04:01/08:01 | 04:06/12:02 |
| 177 | LTG | 11:01/74:02 | 13:01/51:01 | 08:01/14:02 | 03:01/14:04 |
| 178 | LTG | 02:07/2407 | 46:01/35:05 | 01:02/04:01 | 09:01/12:02 |
| 179 | LTG | 11:01/11:01 | 40:01/40:01 | None | None |
| 180 | LTG | 02:03/11:01 | 13:01/13:01 | 03:04/03:04 | 12:02/16:02 |
| 181 | LTG | 02:03/02:07 | 38:02/55:02 | 07:02/12:02 | 08:03/14:54 |
| 182 | LTG | 11:01/24:02 | 13:01/40:01 | 03:04/03:04 | 14:05/15:01 |
| 183 | LTG | 02:07/11:01 | 13:01/46:01 | 01:02/03:04 | 09:01/16:02 |
| 184 | LTG | 11:02/24:02 | 13:01/13:01 | 03:04/03:04 | 15:01/16:02 |
| 185 | LTG | 24:02/33:03 | 35:01/58:01 | 03:02/03:03 | 08:09/13:02 |
| 186 | LTG | 02:07/11:01 | 13:01/13:01 | 03:04/03:17 | 14:05/15:01 |
| 187 | LTG | 24:02/33:03 | 15:02/44:03 | 08:01/14:03 | 13:02/14:54 |
| 188 | LTG | 02:03/02:07 | 13:01/38:02 | 03:04/07:02 | 15:01/16:02 |
| 189 | LTG | 02:07/02:07 | 35:03/46:01 | 01:02/04:01 | 13:01/15:01 |
| 190 | LTG | 02:07/11:01 | 40:01/48:01 | 03:04/14:02 | 14:05/16:02 |
| 191 | LTG | 02:03/02:07 | None | 01:02/03:04 | 09:01/16:02 |
| 192 | LTG | 24:02/24:02 | 13:01/40:01 | 03:04/12:03 | 04:05/15:01 |
| 193 | LTG | 11:01/24:02 | 40:02/51:01 | 03:04/14:02 | 04:05/12:01 |
| 194 | LTG | 02:07/11:01 | 15:02/46:01 | 01:02/08:01 | 09:01/12:02 |
| 195 | LTG | 11:01/11:01 | 13:01/50:01 | 02:02/03:04 | 09:01/15:01 |
| 196 | LTG | 33:03/33:03 | 55:02/58:01 | 03:02/12:02 | 03:01/09:01 |
| 197 | LTG | 02:07/33:03 | 46:01/58:01 | 01:02/03:02 | 03:01/09:01 |
| 198 | LTG | 02:01/33:03 | 40:01/58:01 | 03:02/15:02 | 03:01/11:01 |
| 199 | LTG | 02:01/11:02 | 15:27/54:01 | 01:02/04:01 | 04:05/04:06 |
| 200 | LTG | 01:01/33:03 | 57:01/58:01 | 03:02/06:02 | 13:01/13:02 |
| 201 | LTG | 11:01/24:02 | 15:02/54:01 | 01:02/08:01 | 08:03/15:01 |
| 202 | LTG | 11:01/33:03 | 15:27/58:01 | 03:02/04:01 | 12:02/13:02 |
| 203 | LTG | 11:01/11:01 | 13:01/15:25 | 03:04/04:03 | 12:02/12:02 |
| 204 | LTG | 02:03/11:01 | 40:01/40:01 | 04:03/07:66 | 12:02/15:01 |
| 205 | LTG | 02:03/24:02 | 15:02/48:01 | 08:01/08:03 | 03:01/15:02 |
| 206 | LTG | 02:07/11:01 | 40:01/46:01 | 03:04/07:02 | 09:01/15:02 |
| 207 | LTG | 02:07/02:07 | 13:01/46:01 | 01:02/03:04 | 07:01/15:01 |
| 208 | LTG | 24:02/24:03 | 13:01/15:02 | 03:04/08:01 | 08:03/11:01 |
| 209 | LTG | 02:07/02:07 | 46:01/46:01 | 01:02/01:02 | 09:01/16:02 |
| 210 | LTG | 02:03/24:02 | 35:01/40:01 | 03:04/04:01 | 09:01/14:05 |
| 211 | LTG | 11:02/26:01 | 27:04/38:02 | 07:02/12:02 | 04:05/16:02 |
| 212 | LTG | 02:07/24:02 | 15:02/40:01 | None | None |
| 213 | LTG | 02:06/03:01 | 15:11/2705 | None | None |
| 214 | OXC | 24:02/33:03 | 40:01/40:01 | 03:02/03:04 | 03:01/11:01 |
| 215 | OXC | 02:07/11:01 | 27:04/27:04 | 12:02/14:02 | 11:01/15:02 |
| 216 | OXC | 02:01/30:01 | 46:01/46:01 | 01:02/03:04 | 09:01/14:05 |
| 217 | OXC | 30:01/33:03 | 13:02/35:03 | 04:01/06:02 | 07:01/13:02 |
| 218 | OXC | 02:01/33:03 | 15:27/46:01 | 04:01/14:03 | 04:06/13:02 |
| 219 | OXC | 01:01/11:01 | 40:01/40:01 | 04:01/06:02 | 04:06/15:02 |
| 220 | OXC | 03:01/30:01 | 4901/13:02 | 06:02/07:01 | 04:06/07:01 |
| 221 | OXC | 24:02/24:02 | 40:01/15:27 | 03:04/04:01 | 04:06/16:02 |
| 222 | OXC | 11:01/3101 | 15:01/56:04 | 01:02/03:03 | 14:54/15:02 |
| 223 | OXC | 02:07/11:01 | 15:19/48:04 | 04:03/08:01 | 08:03/11:01 |
| 224 | OXC | 02:03/30:01 | 54:01/13:02 | 06:02/07:02 | 04:05/07:01 |
| 225 | OXC | 02:06/33:03 | 51:01/51:01 | 07:02/14:02 | 12:01/12:02 |
| 226 | OXC | None | 39:05/46:01 | 01:02/07:02 | 04:06/14:54 |
| 227 | OXC | 11:01/11:01 | 15:02/55:02 | 01:02/08:01 | 12:02/15:01 |
| 228 | OXC | 02:07/11:02 | 40:02/46:01 | 01:02/03:04 | 04:03/08:03 |
| 229 | OXC | 02:07/2301 | 45:01/46:01 | 01:02/06:02 | 07:01/08:03 |
| 230 | OXC | 02:03/11:01 | 18:01/46:01 | 07:02/07:04 | 09:01/14:54 |
| 231 | OXC | 02:06/11:02 | 40:02/51:02 | 03:04/15:02 | 04:01/14:54 |
| 232 | OXC | 02:03/02:03 | 38:02/46:01 | 01:02/07:02 | 09:01/16:02 |
| 233 | OXC | 11:01/24:02 | 15:02/40:06 | 03:03/08:01 | 12:01/13:12 |
| 234 | OXC | 11:01/11:01 | 27:07/51:01 | 14:02/15:02 | 07:01/09:01 |
| 235 | OXC | 02:01/02:01 | 15:11/51:01 | 03:03/14:02 | 08:03/12:01 |
| 236 | OXC | 02:07/11:02 | 15:02/51:01 | 08:01/15:02 | 12:02/15:01 |
| 237 | OXC | 02:01/11:01 | 13:01/39:01 | 07:02/07:43 | 11:01/16:02 |
| 238 | OXC | 02:01/11:01 | 15:01/40:01 | 04:01/07:02 | 04:06/08:03 |
| 239 | OXC | 02:03/11:01 | 15:02/38:02 | 07:02/08:01 | 12:02/15:02 |
| 240 | OXC | 02:01/11:01 | 13:01/15:02 | 07:02/08:01 | 12:02/15:01 |
| 241 | OXC | 02:03/33:03 | 40:01/58:01 | 03:02/03:04 | 03:01/15:01 |
| 242 | OXC | 11:01/11:01 | 13:01/35:01 | 03:03/03:04 | 09:01/15:01 |
| 243 | OXC | 02:07/02:07 | 46:01/46:01 | 01:02/01:02 | 09:01/09:01 |
| 244 | OXC | 02:01/11:01 | 15:25/39:01 | 04:03/07:02 | 09:01/16:02 |
| 245 | OXC | 11:01/11:01 | 15:01/35:01 | 04:01/04:01 | 01:01/04:06 |
| 246 | OXC | 02:07/11:01 | 15:02/40:02 | 07:02/08:01 | 04:05/14:54 |
| 247 | OXC | 24:02/3401 | 13:01/40:01 | 07:02/07:02 | 04:05/14:54 |
| 248 | OXC | 24:02/33:03 | 15:01/57:01 | 04:01/06:02 | 07:01/11:01 |
| 249 | OXC | 02:01/33:03 | 40:01/58:01 | 03:02/15:02 | 03:01/11:01 |
| 250 | OXC | 11:01/11:01 | 46:01/46:01 | 01:02/01:02 | 09:01/09:01 |
| 251 | OXC | 26:01/33:03 | 40:01/58:01 | 03:02/07:02 | 03:01/04:05 |
| 252 | OXC | 24:02/26:01 | 48:03/55:02 | 04:03/08:01 | 12:02/15:02 |
| 253 | OXC | 02:03/24:02 | 46:01/51:01 | 01:02/14:02 | 09:01/09:01 |
| 254 | OXC | 01:01/11:01 | 46:01/57:01 | 01:02/06:02 | 07:01/08:03 |
| 255 | OXC | 02:03/02:07 | 13:01/40:01 | 03:04/07:02 | 09:01/12:02 |
| 256 | OXC | 02:03/11:01 | 40:01/40:01 | 04:03/07:66 | 12:02/15:01 |
| 257 | OXC | 02:07/26:01 | 39:01/46:01 | 01:02/07:02 | 08:03/08:03 |
| 258 | OXC | 11:01/11:01 | 15:02/40:01 | 08:01/15:02 | 13:12/15:01 |
| 259 | OXC | 02:03/24:02 | 1512/38:02 | 03:03/07:02 | 04:03/12:02 |
| 260 | OXC | 02:07/02:07 | 13:01/46:01 | 01:02/03:04 | 07:01/15:01 |
| 261 | OXC | 24:02/33:03 | 46:01/58:01 | 01:02/03:02 | 03:01/14:54 |
| 262 | OXC | 02:07/33:03 | 46:01/58:01 | None | None |
| 263 | OXC | None | None | None | 04:05/12:02 |
| 264 | OXC | None | None | None | 12:02/12:02 |
| 265 | OXC | None | None | None | 04:06/16:02 |
| 266 | OXC | None | 13:02/13:02 | None | None |
| 267 | OXC | None | 15:02/27:09 | None | None |

CBZ, carbamazepine; HLA, human leukocyte antigen; LTG, lamotrigine; MPE, maculopapular exanthema; OXC, oxcarbazepine.

**Table S2** Comparison of the presence of all HLA alleles between CBZ-, LTG-, and OXC-induced MPE and the tolerant controls

|  | **No. of HLA genotypes/Total no.(%)** | | |  | | **Cases vs. Tolerant controls** | | |
| --- | --- | --- | --- | --- | --- | --- | --- | --- |
| **Allele** | **MPE^a^** | **Tolerant Controls^a^** |  | | **P-value** | | **OR (95%CI)** | |
| CBZ | | | | | | | | |
| A*01:01 | 1/142(0.70) | 5/178(2.81) |  | | 0.34 | | 0.25(0.03-2.13) | |
| A*02:01 | 14/142(9.86) | 26/178(14.61) |  | | 0.20 | | 0.64(0.32-1.28) | |
| A*02:03 | 25/142(17.61) | 34/178(19.10) |  | | 0.73 | | 0.91(0.51-1.60) | |
| A*02:06 | 10/142(7.04) | 22/178(12.36) |  | | 0.12 | | 0.54(0.25-1.18) | |
| A*02:07 | 39/142(27.46) | 53/178(29.78) |  | | 0.65 | | 0.89(0.55-1.46) | |
| A*02:10 | 1/142(0.70) | 0/178(0.00) |  | | 0.44 | | 2.52(0.23-28.09) | |
| A*03:01 | 3/142(2.11) | 5/178(2.81) |  | | 0.97 | | 0.75(0.18-3.18) | |
| A*11:01 | 72/142(50.70) | 89/178(50.00) |  | | 0.90 | | 1.03(0.66-1.60) | |
| A*11:02 | 10/142(7.04) | 11/178(6.18) |  | | 0.76 | | 1.15(0.47-2.79) | |
| A*11:53 | 0/142(0.00) | 2/178(1.12) |  | | 0.58 | | 0.41(0.04-4.01) | |
| A*24:02 | 32/142(22.54) | 28/178(15.73) |  | | 0.12 | | 1.56(0.89-2.74) | |
| A*24:03 | 1/142(0.70) | 0/178(0.00) |  | | 0.44 | | 2.52(0.23-28.09) | |
| A*24:08 | 0/142(0.00) | 1/178(0.56) |  | | 1.00 | | 0.62(0.06-6.93) | |
| A*24:10 | 1/142(0.70) | 2/178(1.12) |  | | 1.00 | | 0.62(0.06-6.95) | |
| A*24:20 | 1/142(0.70) | 0/178(0.00) |  | | 0.44 | | 2.52(0.23-28.09) | |
| A*26:01 | 5/142(3.52) | 4/178(2.25) |  | | 0.73 | | 1.59(0.42-6.03) | |
| A*29:01 | 0/142(0.00) | 2/178(1.12) |  | | 0.58 | | 0.41(0.04-4.01) | |
| A*30:01 | 8/142(5.63) | 4/178(2.25) |  | | 0.11 | | 2.60(0.77-8.81) | |
| A*31:01 | 10/142(7.04) | 6/178(3.37) |  | | 0.13 | | 2.17(0.77-6.13) | |
| A*31:13 | 0/142(0.00) | 1/178(0.56) |  | | 1.00 | | 0.62(0.06-6.93) | |
| A*32:01 | 1/142(0.70) | 1/178(0.56) |  | | 1.00 | | 1.26(0.08-20.25) | |
| A*33:03 | 19/142(13.38) | 36/178(20.22) |  | | 0.11 | | 0.61(0.33-1.12) | |
| A*68:01 | 1/142(0.70) | 0/178(0.00) |  | | 0.44 | | 2.52(0.23-28.09) | |
| A*74:02 | 2/142(1.41) | 1/178(0.56) |  | | 0.84 | | 2.53(0.23-28.17) | |
| B*07:02 | 1/144(0.69) | 2/179(1.12) |  | | 1.00 | | 0.62(0.06-6.89) | |
| B*07:05 | 0/144(0.00) | 2/179(1.12) |  | | 0.50 | | 0.41(0.04-3.98) | |
| B*08:01 | 1/144(0.69) | 0/179(0.00) |  | | 0.45 | | 2.50(0.22-27.85) | |
| B*13:01 | 33/144(22.92) | 28/179(15.64) |  | | 0.10 | | 1.60(0.92-2.81) | |
| B*13:02 | 10/144(6.94) | 5/179(2.79) |  | | 0.08 | | 2.60(0.87-7.78) | |
| B*15:01 | 11/144(7.64) | 6/179(3.35) |  | | 0.09 | | 2.39(0.86-6.61) | |
| B*15:02 | 22/144(15.28) | 28/179(15.64) |  | | 0.93 | | 0.97(0.53-1.79) | |
| B*15:03 | 0/144(0.00) | 1/179(0.56) |  | | 1.00 | | 0.62(0.06-6.88) | |
| B*15:11 | 1/144(0.69) | 0/179(0.00) |  | | 0.45 | | 2.50(0.22-27.85) | |
| B*15:12 | 1/144(0.69) | 2/179(1.12) |  | | 1.00 | | 0.62(0.06-6.89) | |
| B*15:13 | 0/144(0.00) | 2/179(1.12) |  | | 0.50 | | 0.41(0.04-3.98) | |
| B*15:17 | 1/144(0.69) | 0/179(0.00) |  | | 0.45 | | 2.50(0.22-27.85) | |
| B*15:18 | 1/144(0.69) | 2/179(1.12) |  | | 1.00 | | 0.62(0.06-6.89) | |
| B*15:19 | 2/144(1.39) | 2/179(1.12) |  | | 1.00 | | 1.25(0.17-8.96) | |
| B*15:25 | 3/144(2.08) | 3/179(1.68) |  | | 1.00 | | 1.25(0.25-6.28) | |
| B*15:27 | 3/144(2.08) | 2/179(1.12) |  | | 0.81 | | 1.88(0.31-11.42) | |
| B*15:32 | 0/144(0.00) | 2/179(1.12) |  | | 0.50 | | 0.41(0.04-3.98) | |
| B*18:01 | 0/144(0.00) | 1/179(0.56) |  | | 1.00 | | 0.62(0.06-6.88) | |
| B*18:02 | 2/144(1.39) | 2/179(1.12) |  | | 1.00 | | 1.25(0.17-8.96) | |
| B*27:02 | 1/144(0.69) | 0/179(0.00) |  | | 0.45 | | 2.50(0.22-27.85) | |
| B*27:04 | 5/144(3.47) | 2/179(1.12) |  | | 0.29 | | 3.18(0.61-16.66) | |
| B*35:01 | 7/144(4.86) | 3/179(1.68) |  | | 0.19 | | 3.00(0.76-11.81) | |
| B*35:03 | 1/144(0.69) | 6/179(3.35) |  | | 0.21 | | 0.20(0.02-1.69) | |
| B*35:05 | 1/144(0.69) | 1/179(0.56) |  | | 1.00 | | 1.25(0.08-20.08) | |
| B*37:01 | 0/144(0.00) | 2/179(1.12) |  | | 0.50 | | 0.41(0.04-3.98) | |
| B*38:01 | 1/144(0.69) | 5/179(2.79) |  | | 0.33 | | 0.24(0.03-2.11) | |
| **B*38:02** | **18/144(12.50)** | **10/179(5.59)** |  | | **0.03** | | **2.41(1.08-5.41)** | |
| B*39:01 | 8/144(5.56) | 5/179(2.79) |  | | 0.21 | | 2.05(0.66-6.40) | |
| B*39:05 | 1/144(0.69) | 0/179(0.00) |  | | 0.45 | | 2.50(0.22-27.85) | |
| B*39:09 | 0/144(0.00) | 1/179(0.56) |  | | 1.00 | | 0.62(0.06-6.88) | |
| **B*40:01** | **24/144(16.67)** | **63/179(35.20)** |  | | **1.91×10^-4^** | | | **0.37(0.22-0.63)** |
| B*40:02 | 5/144(3.47) | 2/179(1.12) |  | | 0.29 | | 3.18(0.61-16.66) | |
| B*40:06 | 3/144(2.08) | 2/179(1.12) |  | | 0.81 | | 1.88(0.31-11.42) | |
| B*40:40 | 0/144(0.00) | 1/179(0.56) |  | | 1.00 | | 0.62(0.06-6.88) | |
| B*44:03 | 1/144(0.69) | 2/179(1.12) |  | | 1.00 | | 0.62(0.06-6.89) | |
| B*46:01 | 47/144(32.64) | 47/179(26.26) |  | | 0.21 | | 1.36(0.84-2.20) | |
| B*48:01 | 2/144(1.39) | 2/179(1.12) |  | | 1.00 | | 1.25(0.17-8.96) | |
| B*48:03 | 1/144(0.69) | 3/179(1.68) |  | | 0.77 | | 0.41(0.04-3.99) | |
| B*49:01 | 0/144(0.00) | 1/179(0.56) |  | | 1.00 | | 0.62(0.06-6.88) | |
| B*50:01 | 1/144(0.69) | 1/179(0.56) |  | | 1.00 | | 1.25(0.08-20.08) | |
| B*50:02 | 1/144(0.69) | 0/179(0.00) |  | | 0.45 | | 2.50(0.22-27.85) | |
| B*51:01 | 9/144(6.25) | 8/179(4.47) |  | | 0.48 | | 1.43(0.54-3.79) | |
| B*51:02 | 3/144(2.08) | 4/179(2.23) |  | | 1.00 | | 0.93(0.21-4.23) | |
| B*51:07 | 1/144(0.69) | 0/179(0.00) |  | | 0.45 | | 2.50(0.22-27.85) | |
| B*52:01 | 4/144(2.78) | 1/179(0.56) |  | | 0.25 | | 5.09(0.56-46.01) | |
| B*54:01 | 3/144(2.08) | 6/179(3.35) |  | | 0.73 | | 0.61(0.15-2.50) | |
| B*55:01 | 0/144(0.00) | 2/179(1.12) |  | | 0.50 | | 0.41(0.04-3.98) | |
| B*55:02 | 4/144(2.78) | 10/179(5.59) |  | | 0.22 | | 0.48(0.15-1.57) | |
| B*55:12 | 2/144(1.39) | 0/179(0.00) |  | | 0.20 | | 3.78(0.39-36.69) | |
| B*56:01 | 1/144(0.69) | 5/179(2.79) |  | | 0.33 | | 0.24(0.03-2.11) | |
| B*56:03 | 1/144(0.69) | 2/179(1.12) |  | | 1.00 | | 0.62(0.06-6.89) | |
| B*56:04 | 2/144(1.39) | 1/179(0.56) |  | | 0.85 | | 2.51(0.23-27.93) | |
| B*56:10 | 0/144(0.00) | 1/179(0.56) |  | | 1.00 | | 0.62(0.06-6.88) | |
| B*57:01 | 0/144(0.00) | 1/179(0.56) |  | | 1.00 | | 0.62(0.06-6.88) | |
| B*58:01 | 15/144(10.42) | 26/179(14.53) |  | | 0.27 | | 0.68(0.35-1.35) | |
| B*59:01 | 1/144(0.69) | 1/179(0.56) |  | | 1.00 | | 1.25(0.08-20.08) | |
| B*67:01 | 1/144(0.69) | 0/179(0.00) |  | | 0.45 | | 2.50(0.22-27.85) | |
| C*01:02 | 58/142(40.85) | 61/177(33.9) |  | | 0.24 | | 1.31(0.83-2.07) | |
| C*01:03 | 2/142(1.41) | 1/177(0.56) |  | | 0.85 | | 2.51(0.23-28.01) | |
| C*01:08 | 0/142(0.00) | 1/177(0.56) |  | | 1.00 | | 0.62(0.06-6.89) | |
| C*02:02 | 1/142(0.70) | 1/177(0.56) |  | | 1.00 | | 1.25(0.08-20.13) | |
| C*03:02 | 19/142(13.38) | 34/177(19.21) |  | | 0.17 | | 0.65(0.35-1.20) | |
| C*03:03 | 10/142(7.04) | 11/177(6.21) |  | | 0.77 | | 1.14(0.47-2.77) | |
| C*03:04 | 43/142(30.28) | 44/177(24.86) |  | | 0.28 | | 1.31(0.80-2.15) | |
| C*03:17 | 1/142(0.70) | 1/177(0.56) |  | | 1.00 | | 1.25(0.08-20.13) | |
| C*04:01 | 9/142(6.34) | 8/177(4.52) |  | | 0.47 | | 1.43(0.54-3.81) | |
| C*04:03 | 3/142(2.11) | 7/177(3.95) |  | | 0.54 | | 0.52(0.13-2.07) | |
| C*06:02 | 8/142(5.63) | 9/177(5.08) |  | | 0.83 | | 1.11(0.42-2.97) | |
| C*07:01 | 1/142(0.70) | 2/177(1.13) |  | | 1.00 | | 0.62(0.06-6.91) | |
| C*07:02 | 48/142(33.80) | 57/177(32.20) |  | | 0.76 | | 1.08(0.67-1.72) | |
| C*07:04 | 2/142(1.41) | 6/177(3.39) |  | | 0.45 | | (0.41-0.08-2.05) | |
| C*07:06 | 1/142(0.70) | 1/177(0.56) |  | | 1.00 | | 1.25(0.08-20.13) | |
| C*08:01 | 28/142(19.72) | 38/177(21.47) |  | | 0.70 | | 0.90(0.52-1.55) | |
| C*08:22 | 0/142(0.00) | 2/177(1.13) |  | | 0.50 | | 0.41(0.04-3.99) | |
| C*08:41 | 1/142(0.70) | 0/177(0.00) |  | | 0.45 | | 2.51(0.23-27.93) | |
| C*12:02 | 7/142(4.93) | 7/177(3.95) |  | | 0.67 | | 1.26(0.43-3.68) | |
| **C*12:03** | **2/142(1.41)** | **18/177(10.17)** |  | | **0.001** | | **0.13(0.03-0.55)** | |
| C*14:02 | 9/142(6.34) | 8/177(4.52) |  | | 0.47 | | 1.43(0.54-3.81) | |
| C*14:03 | 1/142(0.70) | 1/177(0.56) |  | | 1.00 | | 1.25(0.08-20.13) | |
| C*15:02 | 10/142(7.04) | 10/177(5.65) |  | | 0.61 | | 1.27(0.51-3.13) | |
| C*15:04 | 2/142(1.41) | 1/177(0.56) |  | | 0.85 | | 2.51(0.23-28.01) | |
| C*15:05 | 2/142(1.41) | 2/177(1.13) |  | | 1.00 | | 1.25(0.17-8.99) | |
| DRB1*01:01 | 2/143(1.40) | 1/176(0.57) |  | | 0.86 | | 2.48(0.22-27.66) | |
| DRB1*03:01 | 16/143(11.19) | 27/176(15.34) |  | | 0.28 | | 0.70(0.36-1.35) | |
| DRB1*04:01 | 0/143(0.00) | 3/176(1.70) |  | | 0.32 | | 0.30(0.03-2.73) | |
| DRB1*04:03 | 4/143(2.80) | 5/176(2.84) |  | | 1.00 | | 0.98(0.26-3.74) | |
| DRB1*04:04 | 3/143(2.10) | 2/176(1.14) |  | | 0.82 | | 1.86(0.31-11.31) | |
| DRB1*04:05 | 10/143(6.99) | 15/176(8.52) |  | | 0.61 | | 0.81(0.35-1.86) | |
| DRB1*04:06 | 7/143(4.90) | 6/176(3.41) |  | | 0.50 | | 1.46(0.48-4.44) | |
| DRB1*07:01 | 11/143(7.69) | 15/176(8.52) |  | | 0.79 | | 0.89(0.40-2.01) | |
| DRB1*08:02 | 0/143(0.00) | 1/176(0.57) |  | | 1.00 | | 0.61(0.06-6.81) | |
| DRB1*08:03 | 16/143(11.19) | 16/176(9.09) |  | | 0.54 | | 1.26(0.61-2.61) | |
| DRB1*08:09 | 0/143(0.00) | 1/176(0.57) |  | | 1.00 | | 0.61(0.06-6.81) | |
| DRB1*08:14 | 0/143(0.00) | 1/176(0.57) |  | | 1.00 | | 0.61(0.06-6.81) | |
| DRB1*09:01 | 36/143(25.17) | 54/176(30.68) |  | | 0.28 | | 0.76(0.46-1.25) | |
| DRB1*10:01 | 0/143(0.00) | 3/176(1.70) |  | | 0.32 | | 0.30(0.03-2.73) | |
| DRB1*11:01 | 12/143(8.39) | 20/176(11.36) |  | | 0.38 | | 0.72(0.34-1.52) | |
| DRB1*11:06 | 0/143(0.00) | 1/176(0.57) |  | | 1.00 | | 0.61(0.06-6.81) | |
| DRB1*12:01 | 4/143(2.80) | 10/176(5.68) |  | | 0.21 | | 0.48(0.15-1.56) | |
| DRB1*12:02 | 44/143(30.77) | 45/176(25.57) |  | | 0.29 | | 1.30(0.80-2.13) | |
| DRB1*12:10 | 0/143(0.00) | 1/176(0.57) |  | | 1.00 | | 0.61(0.06-6.81) | |
| DRB1*13:01 | 0/143(0.00) | 1/176(0.57) |  | | 1.00 | | 0.61(0.06-6.81) | |
| DRB1*13:02 | 5/143(3.50) | 6/176(3.41) |  | | 1.00 | | 1.03(0.31-3.44) | |
| DRB1*13:12 | 3/143(2.10) | 6/176(3.41) |  | | 0.72 | | 0.61(0.15-2.47) | |
| DRB1*14:01 | 1/143(0.70) | 0/176(0.00) |  | | 0.45 | | 2.48(0.22-27.58) | |
| DRB1*14:04 | 3/143(2.10) | 2/176(1.14) |  | | 0.82 | | 1.86(0.31-11.31) | |
| DRB1*14:05 | 8/143(5.59) | 10/176(5.68) |  | | 0.97 | | 0.98(0.38-2.56) | |
| DRB1*14:18 | 3/143(2.10) | 0/176(0.00) |  | | 0.18 | | 5.02(0.56-45.43) | |
| DRB1*14:54 | 10/143(6.99) | 12/176(6.82) |  | | 0.95 | | 1.03(0.43-2.45) | |
| DRB1*15:01 | 34/143(23.78) | 28/176(15.91) |  | | 0.08 | | 1.65(0.94-2.88) | |
| DRB1*15:02 | 10/143(6.99) | 17/176(9.66) |  | | 0.40 | | 0.70(0.31-1.59) | |
| DRB1*15:04 | 0/143(0.00) | 2/176(1.14) |  | | 0.50 | | 0.41(0.04-3.94) | |
| DRB1*16:02 | 24/143(16.78) | 22/176(12.50) |  | | 0.28 | | 1.41(0.76-2.64) | |
| LTG | | | | | | | | |
| A*01:01 | 1/63(1.59) | 1/102(0.98) |  | | 1.00 | | 1.63(0.10-26.52) | |
| A*02:01 | 6/63(9.52) | 11/102(10.78) |  | | 0.80 | | 0.87(0.31-2.48) | |
| A*02:03 | 12/63(19.05) | 15/102(14.71) |  | | 0.46 | | 1.37(0.59-3.14) | |
| A*02:06 | 2/63(3.17) | 13/102(12.75) |  | | 0.04 | | 0.22(0.05-1.03) | |
| A*02:07 | 23/63(36.51) | 28/102(27.45) |  | | 0.22 | | 1.52(0.78-2.98) | |
| A*03:01 | 1/63(1.59) | 1/102(0.98) |  | | 1.00 | | 1.63(0.10-26.52) | |
| A*11:01 | 30/63(47.62) | 58/102(56.86) |  | | 0.25 | | 0.69(0.37-1.30) | |
| A*11:02 | 3/63(4.76) | 8/102(7.84) |  | | 0.65 | | 0.59(0.15-2.30) | |
| A*24:02 | 15/63(23.81) | 16/102(15.69) |  | | 0.19 | | 1.68(0.76-3.69) | |
| A*24:03 | 1/63(1.59) | 0/102(0.00) |  | | 0.38 | | 3.27(0.29-36.80) | |
| A*24:07 | 1/63(1.59) | 1/102(0.98) |  | | 1.00 | | 1.63(0.10-26.52) | |
| A*24:10 | 0/63(0.00) | 1/102(0.98) |  | | 1.00 | | 0.80(0.07-8.97) | |
| A*26:01 | 2/63(3.17) | 7/102(6.86) |  | | 0.51 | | 0.45(0.09-2.21) | |
| A*29:01 | 1/63(1.59) | 2/102(1.96) |  | | 1.00 | | 0.81(0.07-9.08) | |
| A*30:01 | 2/63(3.17) | 1/102(0.98) |  | | 0.67 | | 3.31(0.29-37.29) | |
| A*31:01 | 0/63(0.00) | 1/102(0.98) |  | | 1.00 | | 0.80(0.07-8.97) | |
| A*32:01 | 1/63(1.59) | 2/102(1.96) |  | | 1.00 | | 0.81(0.07-9.08) | |
| A*33:03 | 11/63(17.46) | 21/102(20.59) |  | | 0.62 | | 0.82(0.36-1.83) | |
| A*34:01 | 0/63(0.00) | 1/102(0.98) |  | | 1.00 | | 0.80(0.07-8.97) | |
| A*74:02 | 1/63(1.59) | 0/102(0.00) |  | | 0.38 | | 3.27(0.29-36.80) | |
| B*01:02 | 1/65(1.54) | 0/102(0.00) |  | | 0.39 | | 3.17(0.28-35.66) | |
| B*07:02 | 0/65(0.00) | 1/102(0.98) |  | | 1.00 | | 0.77(0.07-8.69) | |
| B*07:05 | 0/65(0.00) | 2/102(1.96) |  | | 0.52 | | 0.51(0.05-5.01) | |
| B*08:01 | 1/65(1.54) | 1/102(0.98) |  | | 1.00 | | 1.58(0.10-25.68) | |
| B*13:01 | 14/65(21.53) | 24/102(23.53) |  | | 0.77 | | 0.89(0.42-1.88) | |
| B*13:02 | 3/65(4.62) | 3/102(2.94) |  | | 0.89 | | 1.59(0.31-8.16) | |
| B*15:01 | 2/65(3.08) | 2/102(1.96) |  | | 1.00 | | 1.59(0.22-11.56) | |
| B*15:02 | 12/65(18.46) | 19/102(18.63) |  | | 0.98 | | 0.99(0.44-2.20) | |
| B*15:03 | 0/65(0.00) | 1/102(0.98) |  | | 1.00 | | 0.77(0.07-8.69) | |
| B*15:11 | 1/65(1.54) | 2/102(1.96) |  | | 1.00 | | 0.78(0.07-8.79) | |
| B*15:12 | 0/65(0.00) | 1/102(0.98) |  | | 1.00 | | 0.77(0.07-8.69) | |
| B*15:19 | 1/65(1.54) | 2/102(1.96) |  | | 1.00 | | 0.78(0.07-8.79) | |
| B*15:25 | 2/65(3.08) | 1/102(0.98) |  | | 0.69 | | 3.21(0.29-36.09) | |
| B*15:27 | 2/65(3.08) | 2/102(1.96) |  | | 1.00 | | 1.59(0.22-11.56) | |
| B*27:04 | 1/65(1.54) | 2/102(1.96) |  | | 1.00 | | 0.78(0.07-8.79) | |
| B*27:05 | 1/65(1.54) | 0/102(0.00) |  | | 0.39 | | 3.17(0.28-35.66) | |
| B*35:01 | 2/65(3.08) | 1/102(0.98) |  | | 0.69 | | 3.21(0.29-36.09) | |
| B*35:03 | 2/65(3.08) | 1/102(0.98) |  | | 0.69 | | 3.21(0.29-36.09) | |
| B*35:05 | 1/65(1.54) | 2/102(1.96) |  | | 1.00 | | 0.78(0.07-8.79) | |
| B*37:01 | 0/65(0.00) | 1/102(0.98) |  | | 1.00 | | 0.77(0.07-8.69) | |
| B*38:01 | 0/65(0.00) | 1/102(0.98) |  | | 1.00 | | 0.77(0.07-8.69) | |
| B*38:02 | 7/65(10.77) | 4/102(3.92) |  | | 0.16 | | 2.96(0.83-10.54) | |
| B*39:01 | 1/65(1.54) | 2/102(1.96) |  | | 1.00 | | 0.78(0.07-8.79) | |
| B*40:01 | 15/65(23.08) | 21/102(20.59) |  | | 0.70 | | 1.16(0.55-2.45) | |
| B*40:02 | 1/65(1.54) | 2/102(1.96) |  | | 1.00 | | 0.78(0.07-8.79) | |
| B*44:03 | 2/65(3.08) | 4/102(3.92) |  | | 1.00 | | 0.78(0.14-4.37) | |
| B*46:01 | 18/65(27.69) | 30/102(29.41) |  | | 0.81 | | 0.92(0.46-1.83) | |
| B*48:01 | 3/65(4.62) | 1/102(0.98) |  | | 0.33 | | 1.89(0.50-48.03) | |
| B*48:03 | 0/65(0.00) | 2/102(1.96) |  | | 0.52 | | 0.51(0.05-5.01) | |
| B*50:01 | 1/65(1.54) | 1/102(0.98) |  | | 1.00 | | 1.58(0.10-25.68) | |
| B*51:01 | 2/65(3.08) | 9/102(8.82) |  | | 0.25 | | 0.33(0.07-1.57) | |
| B*52:01 | 1/65(1.54) | 1/102(0.98) |  | | 1.00 | | 1.58(0.10-25.68) | |
| B*53:01 | 0/65(0.00) | 1/102(0.98) |  | | 1.00 | | 0.77(0.07-8.69) | |
| B*54:01 | 4/65(6.15) | 5/102(4.90) |  | | 1.00 | | 1.27(0.33-4.92) | |
| B*55:01 | 0/65(0.00) | 4/102(3.92) |  | | 0.27 | | 0.30(0.03-2.63) | |
| B*55:02 | 4/65(6.15) | 4/102(3.92) |  | | 0.77 | | 1.61(0.39-6.66) | |
| B*55:04 | 0/65(0.00) | 1/102(0.98) |  | | 1.00 | | 0.77(0.07-8.69) | |
| B*56:01 | 1/65(1.54) | 0/102(0.00) |  | | 0.39 | | 3.17(0.28-35.66) | |
| B*56:03 | 0/65(0.00) | 1/102(0.98) |  | | 1.00 | | 0.77(0.07-8.69) | |
| B*57:01 | 1/65(1.54) | 0/102(0.00) |  | | 0.39 | | 3.17(0.28-35.66) | |
| B*58:01 | 8/65(12.31) | 18/102(17.65) |  | | 0.35 | | 0.66(0.27-1.61) | |
| B*59:01 | 0/65(0.00) | 1/102(0.98) |  | | 1.00 | | 0.77(0.07-8.69) | |
| B*81:01 | 1/65(1.54) | 0/102(0.00) |  | | 0.39 | | 3.17(0.28-35.66) | |
| C*01:02 | 21/63(33.33) | 35/102(34.31) |  | | 0.90 | | 0.96(0.49-1.86) | |
| C*01:03 | 2/63(3.17) | 2/102(1.96) |  | | 1.00 | | 1.64(0.23-11.94) | |
| C*02:02 | 1/63(1.59) | 1/102(0.98) |  | | 1.00 | | 1.63(0.10-26.52) | |
| C*03:02 | 9/63(14.29) | 22/102(21.57) |  | | 0.25 | | 0.61(0.26-1.42) | |
| C*03:03 | 2/63(3.17) | 6/102(5.88) |  | | 0.68 | | 0.53(0.10-2.68) | |
| C*03:04 | 23/63(36.51) | 27/102(26.47) |  | | 0.17 | | 1.60(0.81-3.14) | |
| C*03:17 | 1/63(1.59) | 1/102(0.98) |  | | 1.00 | | 1.63(0.10-26.52) | |
| C*04:01 | 8/63(12.70) | 8/102(7.84) |  | | 0.31 | | 1.71(0.61-4.81) | |
| C*04:03 | 3/63(4.76) | 4/102(3.92) |  | | 1.00 | | 1.23(0.27-5.66) | |
| C*04:05 | 1/63(1.59) | 0/102(0.00) |  | | 0.38 | | 3.27(0.29-36.80) | |
| C*06:02 | 4/63(6.35) | 4/102(3.92) |  | | 0.74 | | 1.66(0.40-6.89) | |
| C*07:02 | 13/63(20.63) | 28/102(27.45) |  | | 0.33 | | 0.69(0.33-1.45) | |
| C*07:06 | 0/63(0.00) | 1/102(0.98) |  | | 1.00 | | 0.80(0.07-8.97) | |
| C*07:66 | 1/63(1.59) | 0/102(0.00) |  | | 0.38 | | 3.27(0.29-36.80) | |
| C*08:01 | 13/63(20.63) | 24/102(23.53) |  | | 0.67 | | 0.85(0.39-1.81) | |
| C*08:03 | 1/63(1.59) | 0/102(0.00) |  | | 0.38 | | 3.27(0.29-36.80) | |
| C*08:22 | 1/63(1.59) | 1/102(0.98) |  | | 1.00 | | 1.63(0.10-26.52) | |
| C*12:02 | 5/63(7.94) | 6/102(5.88) |  | | 0.85 | | 1.38(0.40-4.72) | |
| C*12:03 | 3/63(4.76) | 7/102(6.86) |  | | 0.83 | | 0.68(0.17-2.73) | |
| C*14:02 | 3/63(4.76) | 10/102(9.8) |  | | 0.38 | | 0.46(0.12-1.74) | |
| C*14:03 | 2/63(3.17) | 1/102(0.98) |  | | 0.67 | | 3.31(0.29-37.29) | |
| C*15:02 | 2/63(3.17) | 5/102(4.90) |  | | 0.89 | | 0.64(0.12-3.38) | |
| C*15:05 | 1/63(1.59) | 2/102(1.96) |  | | 1.00 | | 0.81(0.07-9.08) | |
| DRB1*01:01 | 1/63(1.59) | 2/101(1.98) |  | | 1.00 | | 0.80(0.07-8.99) | |
| DRB1*03:01 | 8/63(12.70) | 14/101(13.86) |  | | 0.83 | | 0.90(0.36-2.30) | |
| DRB1*04:03 | 1/63(1.59) | 2/101(1.98) |  | | 1.00 | | 0.80(0.07-8.99) | |
| DRB1*04:05 | 10/63(15.87) | 12/101(11.88) |  | | 0.47 | | 1.40(0.57-3.46) | |
| DRB1*04:06 | 2/63(3.17) | 2/101(1.98) |  | | 1.00 | | 1.62(0.22-11.82) | |
| DRB1*04:07 | 1/63(1.59) | 0/101(0.00) |  | | 0.38 | | 3.24(0.29-36.45) | |
| DRB1*04:10 | 0/63(0.00) | 1/101(0.99) |  | | 1.00 | | 0.79(0.07-8.88) | |
| DRB1*07:01 | 4/63(6.35) | 6/101(5.94) |  | | 1.00 | | 1.07(0.29-3.96) | |
| DRB1*08:03 | 6/63(9.52) | 6/101(5.94) |  | | 0.58 | | 1.67(0.51-5.42) | |
| DRB1*08:09 | 1/63(1.59) | 3/101(2.97) |  | | 0.97 | | 0.53(0.05-5.18) | |
| DRB1*09:01 | 19/63(30.16) | 28/101(27.72) |  | | 0.74 | | 1.13(0.56-2.25) | |
| DRB1*10:01 | 1/63(1.59) | 3/101(2.97) |  | | 0.97 | | 0.53(0.05-5.18) | |
| DRB1*11:01 | 4/63(6.35) | 9/101(8.91) |  | | 0.77 | | 0.69(0.20-2.35) | |
| DRB1*12:01 | 2/63(3.17) | 6/101(5.94) |  | | 0.67 | | 0.52(0.10-2.66) | |
| DRB1*12:02 | 13/63(20.63) | 22/101(21.78) |  | | 0.86 | | 0.93(0.43-2.02) | |
| DRB1*13:01 | 3/63(4.76) | 0/101(0.00) |  | | 0.11 | | 6.69(0.73-61.22) | |
| DRB1*13:02 | 5/63(7.94) | 8/101(7.92) |  | | 1.00 | | 1.00(0.31-3.21) | |
| DRB1*13:12 | 2/63(3.17) | 2/101(1.98) |  | | 1.00 | | 1.62(0.22-11.82) | |
| DRB1*14:04 | 1/63(1.59) | 1/101(0.99) |  | | 1.00 | | 1.61(0.10-26.26) | |
| DRB1*14:05 | 5/63(7.94) | 6/101(5.94) |  | | 0.86 | | 1.37(0.40-4.67) | |
| DRB1*14:25 | 0/63(0.00) | 1/101(0.99) |  | | 1.00 | | 0.79(0.07-8.88) | |
| DRB1*14:54 | 7/63(11.11) | 12/101(11.88) |  | | 0.88 | | 0.93(0.34-2.50) | |
| DRB1*15:01 | 14/63(22.22) | 21/101(20.79) |  | | 0.83 | | 1.09(0.51-2.34) | |
| DRB1*15:02 | 3/63(4.76) | 10/101(9.90) |  | | 0.38 | | 0.46(0.12-1.72) | |
| DRB1*16:02 | 9/63(14.29) | 16/101(15.84) |  | | 0.79 | | 0.89(0.37-2.15) | |
| OXC | | | | | | | | |
| A*01:01 | 2/48(4.17) | 2/56(3.57) |  | | 1.00 | | 1.17(0.16-8.67) | |
| A*02:01 | 8/48(16.67) | 8/56(14.29) |  | | 0.74 | | 1.20(0.41-3.49) | |
| A*02:03 | 9/48(18.75) | 14/56(25.00) |  | | 0.44 | | 0.69(0.27-1.78) | |
| A*02:06 | 2/48(4.17) | 5/56(8.93) |  | | 0.57 | | 0.44(0.08-2.40) | |
| A*02:07 | 11/48(22.92) | 16/56(28.57) |  | | 0.51 | | 0.74(0.31-1.81) | |
| A*03:01 | 1/48(2.08) | 0/56(0.00) |  | | 0.46 | | 2.38(0.21-27.00) | |
| A*11:01 | 20/48(41.67) | 29/56(51.79) |  | | 0.30 | | 0.67(0.31-1.45) | |
| A*11:02 | 3/48(6.25) | 5/56(8.93) |  | | 0.89 | | 0.68(0.15-3.01) | |
| A*23:01 | 1/48(2.08) | 0/56(0.00) |  | | 0.46 | | 2.38(0.21-27.00) | |
| A*24:02 | 9/48(18.75) | 8/56(14.29) |  | | 0.54 | | 1.39(0.49-3.93) | |
| A*26:01 | 3/48(6.25) | 2/56(3.57) |  | | 0.86 | | 1.80(0.29-11.25) | |
| A*29:01 | 0/48(0.00) | 3/56(5.36) |  | | 0.30 | | 0.28 (0.03-2.55) | |
| A*30:01 | 4/48(8.33) | 2/56(3.57) |  | | 0.54 | | 2.46(0.43-14.03) | |
| A*31:01 | 1/48(2.08) | 2/56(3.57) |  | | 1.00 | | 0.57(0.05-6.54) | |
| A*33:03 | 10/48(20.83) | 9/56(16.07) |  | | 0.53 | | 1.37(0.51-3.72) | |
| A*34:01 | 1/48(2.08) | 1/56(1.79) |  | | 1.00 | | 1.17(0.07-19.23) | |
| B*07:02 | 0/51(0) | 1/91(1.10) |  | | 1.00 | | 0.88(0.08-9.89) | |
| B*07:05 | 0/51(0) | 4/91(4.40) |  | | 0.32 | | 0.34(0.04-2.98) | |
| B*13:01 | 6/51(11.76) | 9/91(9.89) |  | | 0.73 | | 1.22(0.41-3.63) | |
| B*13:02 | 4/51(7.84) | 3/91(3.30) |  | | 0.43 | | 2.50(0.54-11.63) | |
| B*15:01 | 4/51(7.84) | 5/91(5.49) |  | | 0.85 | | 1.46(0.38-5.72) | |
| B*15:02 | 8/51(15.69) | 9/91(9.89) |  | | 0.31 | | 1.70(0.61-4.71) | |
| B*15:11 | 1/51(1.96) | 1/91(1.10) |  | | 1.00 | | 1.80(0.11-29.40) | |
| B*15:12 | 1/51(1.96) | 1/91(1.10) |  | | 1.00 | | 1.80(0.11-29.40) | |
| B*15:19 | 1/51(1.96) | 1/91(1.10) |  | | 1.00 | | 1.80(0.11-29.40) | |
| B*15:25 | 1/51(1.96) | 2/91(2.20) |  | | 1.00 | | 0.89(0.08-10.06) | |
| B*15:27 | 2/49(4.08) | 0/56(0.00) |  | | 0.13 | | 5.52(0.56-54.47) | |
| B*15:35 | 0/51(0) | 1/91(1.10) |  | | 1.00 | | 0.88(0.08-9.89) | |
| B*18:01 | 1/51(1.96) | 0/91(0.00) |  | | 0.36 | | 3.61(0.32-40.77) | |
| B*27:04 | 1/51(1.96) | 0/91(0.00) |  | | 0.36 | | 3.61(0.32-40.77) | |
| B*27:07 | 1/51(1.96) | 0/91(0.00) |  | | 0.36 | | 3.61(0.32-40.77) | |
| B*27:09 | 1/51(1.96) | 0/91(0.00) |  | | 0.36 | | 3.61(0.32-40.77) | |
| B*35:01 | 2/51(3.92) | 1/91(1.10) |  | | 0.61 | | 3.67(0.33-41.54) | |
| B*35:03 | 1/51(1.96) | 1/91(1.10) |  | | 1.00 | | 1.80(0.11-29.40) | |
| B*38:02 | 3/51(5.88) | 9/91(9.89) |  | | 0.61 | | 0.57(0.15-2.21) | |
| B*39:01 | 3/51(5.88) | 1/91(1.10) |  | | 0.26 | | 5.63(0.57-55.55) | |
| B*39:05 | 1/51(1.96) | 0/91(0.00) |  | | 0.36 | | 3.61(0.32-40.77) | |
| B*40:01 | 11/51(21.57) | 31/91(34.07) |  | | 0.12 | | 0.53(0.24-1.18) | |
| B*40:02 | 3/51(5.88) | 2/91(2.20) |  | | 0.50 | | 2.78(0.45-17.22) | |
| B*40:06 | 1/51(1.96) | 0/91(0.00) |  | | 0.36 | | 3.61(0.32-40.77) | |
| B*45:01 | 1/51(1.96) | 0/91(0.00) |  | | 0.36 | | 3.61(0.32-40.77) | |
| B*46:01 | 15/51(29.41) | 27/91(29.67) |  | | 0.97 | | 0.99(0.47-2.09) | |
| B*48:03 | 1/51(1.96) | 2/91(2.20) |  | | 1.00 | | 0.89(0.08-10.06) | |
| B*48:04 | 1/51(1.96) | 0/91(0.00) |  | | 0.36 | | 3.61(0.32-40.77) | |
| B*49:01 | 1/51(1.96) | 0/91(0.00) |  | | 0.36 | | 3.61(0.32-40.77) | |
| B*50:01 | 0/51(0) | 1/91(1.10) |  | | 1.00 | | 0.88(0.08-9.89) | |
| B*51:01 | 5/51(9.80) | 8/91(8.79) |  | | 1.00 | | 1.13(0.35-3.65) | |
| B*54:01 | 1/51(1.96) | 0/91(0.00) |  | | 0.36 | | 3.61(0.32-40.77) | |
| B*55:01 | 1/51(1.96) | 1/91(1.10) |  | | 1.00 | | 1.80(0.11-29.40) | |
| B*55:02 | 0/51(0) | 1/91(1.10) |  | | 1.00 | | 0.88(0.08-9.89) | |
| B*56:04 | 2/51(3.92) | 4/91(4.40) |  | | 1.00 | | 0.89(0.16-5.02) | |
| B*57:01 | 1/51(1.96) | 0/91(0.00) |  | | 0.36 | | 3.61(0.32-40.77) | |
| B*58:01 | 2/51(3.92) | 0/91(0.00) |  | | 0.13 | | 5.52(0.56-54.47) | |
| C*01:02 | 14/48(29.17) | 17/52(32.69) |  | | 0.70 | | 0.85(0.36-1.98) | |
| C*02:02 | 0/48(0.00) | 1/52(1.92) |  | | 1.00 | | 0.53(0.05-6.04) | |
| C*03:02 | 5/48(10.42) | 8/52(15.38) |  | | 0.55 | | 0.70(0.21-2.30) | |
| C*03:03 | 5/48(10.42) | 1/52(1.92) |  | | 0.17 | | 5.93(0.67-52.73) | |
| C*03:04 | 9/48(18.75) | 14/52(26.92) |  | | 0.33 | | 0.63(0.24-1.62) | |
| C*04:01 | 7/48(14.58) | 4/52(7.69) |  | | 0.27 | | 2.05(0.56-7.50) | |
| C*04:03 | 4/48(8.33) | 3/52(5.77) |  | | 0.91 | | 1.49(0.32-7.01) | |
| C*06:02 | 7/48(14.58) | 2/52(3.85) |  | | 0.13 | | 4.27(0.84-21.67) | |
| C*07:02 | 16/48(33.33) | 15/52(28.85) |  | | 0.63 | | 1.23 (0.53-2.88) | |
| C*07:43 | 1/48(2.08) | 0/52(0.00) |  | | 0.48 | | 2.21(0.19-25.14) | |
| C*07:66 | 1/48(2.08) | 0/52(0.00) |  | | 0.48 | | 2.21(0.19-25.14) | |
| C*08:01 | 9/48(18.75) | 9/52(17.31) |  | | 0.85 | | 1.10(0.40-3.06) | |
| C*12:02 | 1/48(2.08) | 2/52(3.85) |  | | 1.00 | | 0.53(0.05-6.06) | |
| C*12:03 | 0/48(0.00) | 3/52(5.77) |  | | 0.27 | | 0.26(0.03-2.36) | |
| C*14:02 | 5/48(10.42) | 2/52(3.85) |  | | 0.37 | | 2.91(0.54-15.75) | |
| C*14:03 | 1/48(2.08) | 0/52(0.00) |  | | 0.48 | | 2.21(0.19-25.14) | |
| C*15:02 | 5/48(10.42) | 7/52(13.46) |  | | 0.64 | | 0.75(0.22-2.54) | |
| C*15:05 | 0/48(0.00) | 3/52(5.77) |  | | 0.27 | | 0.26(0.03-2.36) | |
| DRB1*01:01 | 1/51(1.96) | 2/94(2.13) |  | | 1.00 | | 0.92(0.08-10.40) | |
| DRB1*03:01 | 5/51(9.80) | 9/94(9.57) |  | | 1.00 | | 1.03(0.33-3.24) | |
| DRB1*04:03 | 2/51(3.92) | 8/94(8.51) |  | | 0.49 | | 0.44(0.09-2.15) | |
| DRB1*04:05 | 5/51(9.80) | 6/94(6.38) |  | | 0.68 | | 1.59(0.446-5.51) | |
| **DRB1*04:06** | **8/51(15.69)** | **1/94(1.06)** |  | | **0.002** | | **17.30(2.10-142.72)** | |
| DRB1*07:01 | 8/51(15.69) | 5/94(5.32) |  | | 0.08 | | 3.31(1.02-10.73) | |
| DRB1*08:03 | 7/51(13.73) | 15/94(15.94) |  | | 0.72 | | 0.84(00.32-2.21) | |
| DRB1*09:01 | 10/51(19.61) | 33/94(35.11) |  | | 0.05 | | 0.45(0.20-1.01) | |
| DRB1*10:01 | 0/51(0.00) | 4/94(4.26) |  | | 0.34 | | 0.35(0.04-3.08) | |
| DRB1*11:01 | 6/51(11.76) | 9/94(9.57) |  | | 0.68 | | 1.26(0.42-3.76) | |
| DRB1*12:01 | 3/51(5.88) | 3/94(3.19) |  | | 0.73 | | 1.90(0.37-9.75) | |
| DRB1*12:02 | 11/51(21.57) | 19/94(20.21) |  | | 0.85 | | 1.09(0.47-2.50) | |
| DRB1*12:10 | 0/51(0.00) | 1/94(1.06) |  | | 1.00 | | 0.90(0.08-10.21) | |
| DRB1*13:02 | 2/51(3.92) | 2/94(2.13) |  | | 0.92 | | 1.88(0.26-13.74) | |
| DRB1*13:12 | 2/51(3.92) | 3/94(3.19) |  | | 1.00 | | 1.24(0.20-7.66) | |
| DRB1*14:05 | 1/51(1.96) | 3/94(3.19) |  | | 1.00 | | 0.61(0.06-5.99) | |
| DRB1*14:54 | 7/51(13.73) | 8/94(8.51) |  | | 0.33 | | 1.71(0.58-5.02) | |
| DRB1*15:01 | 8/51(15.69) | 19/94(20.21) |  | | 0.50 | | 0.73(0.30-1.82) | |
| DRB1*15:02 | 5/51(9.80) | 11/94(11.70) |  | | 0.73 | | 0.82(0.27-2.51) | |
| DRB1*16:02 | 5/51(9.80) | 16/94(17.02) |  | | 0.24 | | 0.53(0.18-1.54) | |
| Pooled | | | | | | | | |
| A*01:01 | 4/253(1.58) | 6/308(1.95) |  | | 1.00 | | 0.81(0.23-2.90) | |
| A*02:01 | 28/253(11.07) | 40/308(12.99) |  | | 0.49 | | 0.83(0.50-1.40) | |
| A*02:03 | 46/253(18.18) | 57/308(18.51) |  | | 0.92 | | 0.98(0.64-1.50) | |
| A*02:06 | 14/253(5.53) | 38/308(12.34) |  | | 0.006 | | 0.42(0.22-0.79) | |
| A*02:07 | 73/253(28.85) | 90/308(29.22) |  | | 0.92 | | 0.98(0.68-1.42) | |
| A*02:10 | 1/253(0.40) | 0/308(0.00) |  | | 0.45 | | 2.44 (0.22-27.09) | |
| A*03:01 | 5/253(1.98) | 6/308(1.95) |  | | 1.00 | | 1.02(0.31-3.37) | |
| A*11:01 | 122/253(48.22) | 160/308(51.95) |  | | 0.38 | | 0.86(0.62-1.20) | |
| A*11:02 | 16/253(6.32) | 22/308(7.14) |  | | 0.70 | | 0.88(0.45-1.71) | |
| **A*24:02** | **56/253(22.13)** | **48/308(15.58)** |  | | **0.047** | | **1.54(1.00-2.36)** | |
| A*24:07 | 1/253(0.40) | 3/308(0.97) |  | | 0.76 | | 0.40(0.04-3.90) | |
| A*24:10 | 1/253(0.40) | 3/308(0.97) |  | | 0.76 | | 0.40(0.04-3.90) | |
| A*24:20 | 1/253(0.40) | 0/308(0.00) |  | | 0.45 | | 2.44 (0.22-27.09) | |
| A*26:01 | 10/253(3.95) | 13/308(4.22) |  | | 0.87 | | 0.93(0.40-2.17) | |
| A*29:01 | 1/253(0.40) | 4/308(1.30) |  | | 0.50 | | 0.30 (0.03-2.72) | |
| **A*30:01** | **14/253(5.53)** | **7/308(2.27)** |  | | **0.043** | | **2.52(1.00-6.34)** | |
| A*31:01 | 11/253(4.35) | 8/308(2.60) |  | | 0.25 | | 1.71(0.68-4.30) | |
| A*32:01 | 2/253(0.79) | 3/308(0.97) |  | | 1.00 | | 0.81(0.13-4.89) | |
| A*33:03 | 40/253(15.81) | 61/308(19.81) |  | | 0.22 | | 0.76(0.49-1.18) | |
| A*34:01 | 1/253(0.40) | 1/308(0.32) |  | | 1.00 | | 1.22(0.08-19.58) | |
| A*68:01 | 1/253(0.40) | 0/308(0.00) |  | | 0.45 | | 2.44 (0.22-27.09) | |
| A*74:02 | 3/253(1.19) | 1/308(0.32) |  | | 0.48 | | 3.68(0.38-35.64) | |
| B*07:02 | 1/260(0.38) | 3/344(0.87) |  | | 0.82 | | 0.44(0.05-4.24) | |
| B*07:05 | 0/260(0.00) | 5/344(1.45) |  | | 0.13 | | 0.22(0.03-1.81) | |
| B*08:01 | 2/260(0.77) | 2/344(0.58) |  | | 1.00 | | 1.33(0.19-9.47) | |
| B*13:01 | 53/260(20.38) | 57/344(16.57) |  | | 0.23 | | 1.29(0.85-1.95) | |
| B*13:02 | 17/260(6.54) | 13/344(3.78) |  | | 0.12 | | 1.78(0.85-3.74) | |
| B*15:01 | 17/260(6.54) | 13/344(3.78) |  | | 0.12 | | 1.78(0.85-3.74) | |
| B*15:02 | 42/260(16.15) | 50/344(14.53) |  | | 0.58 | | 1.13(0.73-1.77) | |
| B*15:03 | 0/260(0.00) | 2/344(0.58) |  | | 0.51 | | 0.44(0.05-4.24) | |
| B*15:11 | 3/260(1.15) | 3/344(0.87) |  | | 1.00 | | 1.33(0.27-6.63) | |
| B*15:12 | 2/260(0.77) | 3/344(0.87) |  | | 1.00 | | 0.88(0.15-5.31) | |
| B*15:13 | 1/260(0.38) | 2/344(0.58) |  | | 1.00 | | 0.66(0.06-7.32) | |
| B*15:18 | 1/260(0.38) | 2/344(0.58) |  | | 1.00 | | 0.66(0.06-7.32) | |
| B*15:19 | 4/260(1.54) | 4/344(1.16) |  | | 0.97 | | 1.33(0.33-5.36) | |
| B*15:25 | 6/260(2.31) | 6/344(1.74) |  | | 0.62 | | 1.33(0.42-4.17) | |
| B*15:27 | 7/260(2.69) | 4/344(1.16) |  | | 0.28 | | 2.35(0.68-8.12) | |
| B*18:01 | 1/260(0.38) | 1/344(0.29) |  | | 1.00 | | 1.32(0.08-21.27) | |
| B*18:02 | 2/260(0.77) | 2/344(0.58) |  | | 1.00 | | 1.33(0.19-9.47) | |
| B*27:04 | 7/260(2.69) | 4/344(1.16) |  | | 0.28 | | 2.35(0.68-8.12) | |
| **B*35:01** | **11/260(4.23)** | **4/344(1.16)** |  | | **0.02** | | **3.76(1.18-11.93)** | |
| B*35:03 | 4/260(1.54) | 7/344(2.03) |  | | 0.89 | | 0.75(0.22-2.60) | |
| B*35:05 | 2/260(0.77) | 7/344(2.03) |  | | 0.35 | | 0.37(0.08-1.81) | |
| B*37:01 | 0/260(0.00) | 3/344(0.87) |  | | 0.36 | | 0.33(0.04-2.95) | |
| B*38:01 | 1/260(0.38) | 6/344(1.74) |  | | 0.25 | | 0.22(0.03-1.82) | |
| **B*38:02** | **28/260(10.77)** | **20/344(5.81)** |  | | **0.03** | | **1.96(1.08-3.56)** | |
| B*39:01 | 12/260(4.62) | 7/344(2.03) |  | | 0.07 | | 2.32(0.90-6.00) | |
| B*39:05 | 2/260(0.77) | 0/344(0.00) |  | | 0.19 | | 4.00(0.41-38.64) | |
| **B*40:01** | **50/260(19.23)** | **106/344(30.81)** |  | | **0.001** | | **0.54(0.36-0.79)** | |
| B*40:02 | 9/260(3.46) | 6/344(1.74) |  | | 0.18 | | 2.02(0.71-5.75) | |
| B*40:06 | 3/260(1.15) | 2/344(0.58) |  | | 0.75 | | 2.00(0.33-12.03) | |
| B*44:03 | 3/260(1.15) | 6/344(1.74) |  | | 0.80 | | 0.66(0.16-2.65) | |
| B*46:01 | 80/260(30.77) | 94/344(27.33) |  | | 0.36 | | 1.18(0.83-1.69) | |
| B*48:01 | 5/260(1.92) | 6/344(1.74) |  | | 1.00 | | 1.11(0.33-3.66) | |
| B*48:03 | 2/260(0.77) | 7/344(2.03) |  | | 0.35 | | 0.37(0.08-1.81) | |
| B*49:01 | 1/260(0.38) | 1/344(0.29) |  | | 1.00 | | 1.32(0.08-21.27) | |
| B*50:01 | 2/260(0.77) | 2/344(0.58) |  | | 1.00 | | 1.33(0.19-9.47) | |
| B*51:01 | 16/260(6.15) | 22/344(6.40) |  | | 0.90 | | 0.96(0.49-1.87) | |
| B*51:02 | 4/260(1.54) | 4/344(1.16) |  | | 0.97 | | 1.33(0.33-5.36) | |
| B*51:07 | 1/260(0.38) | 0/344(0.00) |  | | 0.43 | | 2.65(0.24-29.43) | |
| B*52:01 | 5/260(1.92) | 5/344(1.45) |  | | 0.90 | | 1.33(0.38-4.64) | |
| B*54:01 | 8/260(3.08) | 12/344(3.49) |  | | 0.78 | | 0.88(0.35-2.18) | |
| B*55:02 | 10/260(3.85) | 18/344(5.23) |  | | 0.42 | | 0.72(0.33-1.60) | |
| B*55:12 | 2/260(0.77) | 0/344(0.00) |  | | 0.19 | | 4.00(0.41-38.64) | |
| B*56:01 | 2/260(0.77) | 5/344(1.45) |  | | 0.69 | | 0.53(0.10-2.73) | |
| B*56:03 | 1/260(0.38) | 5/344(1.45) |  | | 0.37 | | 0.26(0.03-2.25) | |
| B*56:04 | 3/260(1.15) | 1/344(0.29) |  | | 0.43 | | 4.00(0.41-38.71) | |
| B*57:01 | 3/260(1.15) | 1/344(0.29) |  | | 0.43 | | 4.00(0.41-38.71) | |
| B*58:01 | 28/260(10.77) | 50/344(14.53) |  | | 0.17 | | 0.71(0.43-1.16) | |
| B*59:01 | 1/260(0.38) | 2/344(0.58) |  | | 1.00 | | 0.66(0.06-7.32) | |
| B*67:01 | 1/260(0.38) | 0/344(0.00) |  | | 0.43 | | 2.65(0.24-29.43) | |
| B*81:01 | 1/260(0.38) | 0/344(0.00) |  | | 0.43 | | 2.65(0.24-29.43) | |
| C*01:02 | 93/253(36.76) | 104/303(34.32) |  | | 0.55 | | 1.11(0.79-1.58) | |
| C*01:03 | 4/253(1.58) | 3/303(0.99) |  | | 0.81 | | 1.61(0.36-7.25) | |
| C*02:02 | 2/253(0.79) | 2/303(0.66) |  | | 1.00 | | 1.20(0.17-8.57) | |
| C*03:02 | 33/253(13.04) | 60/303(19.80) |  | | 0.03 | | 0.61(0.38-0.97) | |
| C*03:03 | 17/253(6.72) | 17/303(5.61) |  | | 0.59 | | 1.21(0.61-2.43) | |
| C*03:04 | 75/253(29.64) | 77/303(25.41) |  | | 0.27 | | 1.24(0.85-1.80) | |
| C*03:17 | 2/253(0.79) | 2/303(0.66) |  | | 1.00 | | 1.20(0.17-8.57) | |
| C*04:01 | 24/253(9.49) | 19/303(6.27) |  | | 0.16 | | 1.57(0.84-2.93) | |
| C*04:03 | 10/253(3.95) | 12/303(3.96) |  | | 1.00 | | 1.00(0.42-2.35) | |
| C*04:05 | 1/253(0.40) | 0/303(0.00) |  | | 0.46 | | 2.40(0.22-26.66) | |
| C*06:02 | 19/253(7.51) | 15/303(4.95) |  | | 0.21 | | 1.56(0.78-3.14) | |
| C*07:01 | 1/253(0.40) | 2/303(0.66) |  | | 1.00 | | 0.66(0.06-7.32) | |
| C*07:02 | 77/253(30.43) | 93/303(30.69) |  | | 0.95 | | 0.99(0.69-1.42) | |
| C*07:04 | 2/253(0.79) | 6/303(1.98) |  | | 0.42 | | 0.39(0.08-1.97) | |
| C*07:06 | 1/253(0.40) | 2/303(0.66) |  | | 1.00 | | 0.66(0.06-7.32) | |
| C*07:66 | 2/253(0.79) | 0/303(0.00) |  | | 0.21 | | 3.62(0.37-35.01) | |
| C*08:01 | 50/253(19.76) | 64/303(21.12) |  | | 0.69 | | 0.92(0.61-1.39) | |
| C*08:03 | 1/253(0.40) | 0/303(0.00) |  | | 0.46 | | 2.40(0.22-26.66) | |
| C*08:22 | 1/253(0.40) | 3/303(0.99) |  | | 0.75 | | 0.40(0.04-3.84) | |
| C*08:41 | 1/253(0.40) | 0/303(0.00) |  | | 0.46 | | 2.40(0.22-26.66) | |
| C*12:02 | 13/253(5.14) | 14/303(4.62) |  | | 0.78 | | 1.12(0.52-2.43) | |
| **C*12:03** | **5/253(1.98)** | **26/303(8.58)** |  | | **0.001** | | **0.22(0.08-0.57)** | |
| C*14:02 | 17/253(6.72) | 19/303(6.27) |  | | 0.83 | | 1.08(0.55-2.12) | |
| C*14:03 | 4/253(1.58) | 2/303(0.66) |  | | 0.53 | | 2.42(0.44-13.31) | |
| C*15:02 | 17/253(6.72) | 19/303(6.27) |  | | 0.83 | | 1.08(0.55-2.12) | |
| C*15:04 | 2/253(0.79) | 1/303(0.33) |  | | 0.88 | | 2.41(0.22-26.69) | |
| C*15:05 | 3/253(1.19) | 4/303(1.32) |  | | 1.00 | | 0.90(0.20-4.05) | |
| DRB1*01:01 | 4/257(1.56) | 4/343(1.17) |  | | 0.96 | | 1.34(0.33-5.41) | |
| DRB1*03:01 | 29/257(11.28) | 45/343(13.12) |  | | 0.50 | | 0.84(0.51-1.39) | |
| DRB1*04:03 | 7/257(2.72) | 13/343(3.79) |  | | 0.47 | | 0.71(0.28-1.81) | |
| DRB1*04:04 | 3/257(1.17) | 2/343(0.58) |  | | 0.75 | | 2.01(0.33-12.14) | |
| DRB1*04:05 | 25/257(9.73) | 32/343(9.33) |  | | 0.87 | | 1.05(0.60-1.82) | |
| **DRB1*04:06** | **17/257(6.61)** | **9/343(2.62)** |  | | **0.02** | | **2.63(1.15-6.00)** | |
| DRB1*04:07 | 1/257(0.39) | 0/343(0.00) |  | | 0.43 | | 2.68(0.24-29.68) | |
| DRB1*07:01 | 23/257(8.95) | 25/343(7.29) |  | | 0.46 | | 1.25(0.69-2.26) | |
| DRB1*08:03 | 29/257(11.28) | 36/343(10.50) |  | | 0.76 | | 1.09(0.65-1.82) | |
| DRB1*08:09 | 1/257(0.39) | 4/343(1.17) |  | | 0.56 | | 0.33(0.04-2.98) | |
| DRB1*09:01 | 65/257(25.29) | 106/343(30.90) |  | | 0.13 | | 0.76(0.53-1.09) | |
| DRB1*10:01 | 1/257(0.39) | 7/343(2.04) |  | | 0.17 | | 0.19(0.02-1.53) | |
| DRB1*11:01 | 22/257(8.56) | 36/343(10.50) |  | | 0.43 | | 0.80(0.46-1.39) | |
| DRB1*12:01 | 9/257(3.50) | 18/343(5.25) |  | | 0.31 | | 0.66(0.29-1.48) | |
| DRB1*12:02 | 68/257(26.46) | 82/343(23.91) |  | | 0.48 | | 1.15(0.79-1.66) | |
| DRB1*13:01 | 3/257(1.17) | 1/343(0.87) |  | | 0.43 | | 4.04(0.42-39.06) | |
| DRB1*13:02 | 12/257(4.67) | 16/343(4.66) |  | | 1.00 | | 1.00(0.47-2.16) | |
| DRB1*13:12 | 7/257(2.72) | 11/343(3.21) |  | | 0.73 | | 0.85(0.32-2.21) | |
| DRB1*14:04 | 4/257(1.56) | 4/343(1.17) |  | | 0.96 | | 1.34(0.33-5.41) | |
| DRB1*14:05 | 14/257(5.45) | 17/343(4.96) |  | | 0.79 | | 1.11(0.53-2.29) | |
| DRB1*14:18 | 3/257(1.17) | 0/343(0.00) |  | | 0.16 | | 5.40(0.60-48.57) | |
| DRB1*14:54 | 24/257(9.34) | 28/343(8.16) |  | | 0.61 | | 1.16(0.66-2.05) | |
| DRB1*15:01 | 56/257(21.79) | 63/343(18.37) |  | | 0.30 | | 1.24(0.83-1.85) | |
| DRB1*15:02 | 18/257(7.00) | 33/343(9.62) |  | | 0.26 | | 0.71(0.39-1.29) | |
| DRB1*16:02 | 38/257(14.79) | 50/343(14.58) |  | | 0.94 | | 1.02(0.64-1.61) | |

CBZ, carbamazepine; HLA, human leukocyte antigen; LTG, lamotrigine; MPE, maculopapular exanthema; OXC, oxcarbazepine.

^a^ Several individuals were not subjected to HLA genotyping because of insufficient DNA.

Black bold text indicated the alleles showing positive significant association with CBZ-, LTG-, and OXC-induced MPE and suggested a possible risk role; Blue bold text indicated the alleles showing negative significant association with CBZ-, LTG-, and OXC-induced MPE and suggested a possible protective role.

**Table S3** HLA-A genotypes in patients with AEDs-induced MPE reported by Wang et al. 2014 (provided by the corresponding author)

| **Patient no.** | **Culprit drug** | **HLA-A genotype** |  | | **Patient no.** | **Culprit drug** | | **HLA-A genotype** | |  |
| --- | --- | --- | --- | --- | --- | --- | --- | --- | --- | --- |
| 1 | CBZ | 0201/0207 | | 20 | | | LTG | | 1101/3001 | |
| 2 | CBZ | 1102/2601 | | 21 | | | LTG | | 2402/3001 | |
| 3 | CBZ | 0207/3303 | | 22 | | | LTG | | 0207/2402 | |
| 4 | CBZ | 2402/3303 | | 23 | | | LTG | | 0201/0207 | |
| 5 | CBZ | 1102/1102 | | 24 | | | LTG | | 0207/1101 | |
| 6 | CBZ | 0201/2402 | | 25 | | | LTG | | 0207/1101 | |
| 7 | CBZ | 0201/2402 | | 26 | | | LTG | | 1101/1102 | |
| 8 | CBZ | 1101/3303 | | 27 | | | LTG | | 1101/1101 | |
| 9 | CBZ | 1101/1101 | | 28 | | | LTG | | 2402/3201 | |
| 10 | CBZ | 0206/1101 | | 29 | | | LTG | | 1101/1101 | |
| 11 | CBZ | 0101/1101 | | 30 | | | LTG | | 3101/3303 | |
| 12 | CBZ | 1101/2402 | | 31 | | | LTG | | 1101/2402 | |
| 13 | LTG | 0207/3001 | | 32 | | | LTG | | 1101/2402 | |
| 14 | LTG | 2601/3101 | | 33 | | | LTG | | 0207/3303 | |
| 15 | LTG | 0207/1101 | | 34 | | | LTG | | 0207/1101 | |
| 16 | LTG | 2402/2402 | | 35 | | | LTG | | 3101/3303 | |
| 17 | LTG | 2402/3303 | | 36 | | | LTG | | 2402/3201 | |
| 18 | LTG | 0207/1102 | | 37 | | | LTG | | 1101/1101 | |
| 19 | LTG | 0207/2901 | | 38 | | | PHT | | 0207/1101 | |

CBZ, carbamazepine; HLA, human leukocyte antigen; LTG, lamotrigine; MPE, maculopapular exanthema; OXC, oxcarbazepine; PHT, phenytoin.

Reference: Wang W, Hu FY, Wu XT, et al. Genetic susceptibility to the cross-reactivity of aromatic antiepileptic drugs-induced cutaneous adverse reactions. [Epilepsy Res.](http://www.ncbi.nlm.nih.gov/pubmed/?term=Genetic+susceptibility+to+the+cross-reactivity+of+aromatic+antiepilepticdrugs-induced+cutaneous+adverse+reactions) 2014;108(6):1041-1045.

**Table S4** HLA-A genotypes in Malaysia patients with AEDs-induced MPE (unpublished, provided by Lim et al.)

| **Patient no.** | **Culprit drug** | **HLA-A genotype** | **Patient no.** | **Culprit drug** | **HLA-A genotype** |
| --- | --- | --- | --- | --- | --- |
|
| 1 | CBZ | 0101/0211 | 16 | CBZ | 0207/1101 |
| 2 | CBZ | 0201/3303 | 17 | CBZ | 2402/2402 |
| 3 | CBZ | 0203/0301 | 18 | LTG | 0207/3201 |
| 4 | CBZ | 0203/2402 | 19 | LTG | 1101/3303 |
| 5 | CBZ | 2402/2402 | 20 | LTG | 2402/3401 |
| 6 | CBZ | 0207/1101 | 21 | LTG | 1102/1119 |
| 7 | CBZ | 1101/3101 | 22 | LTG | 2301/2402 |
| 8 | CBZ | 0213/6801 | 23 | LTG | 0206/1104 |
| 9 | CBZ | 2402/3101 | 24 | LTG | 0203/1101 |
| 10 | CBZ | 1101/0201 | 25 | LTG | 2402/2402 |
| 11 | CBZ | 0207/1110 | 26 | LTG | 0211/2601 |
| 12 | CBZ | 0203/3303 | 27 | CBZ | 2402/2402 |
| 13 | CBZ | 0201/2601 | 28 | PHT | 2402/3303 |
| 14 | CBZ | 2402/7413 | 29 | PHT | 0207/2402 |
| 15 | CBZ | 2402/3201 | 30 | CBZ | 2402/3201 |

CBZ, carbamazepine; HLA, human leukocyte antigen; LTG, lamotrigine; MPE, maculopapular exanthema; OXC, oxcarbazepine; PHT, phenytoin.
