# Supplementary figures and images for: HLA Risk Alleles in Aromatic Antiepileptic Drug-Induced Maculopapular Exanthema
Source: Front Pharmacol. 2021 May 26;12:671572. doi: 10.3389/fphar.2021.671572 (PMC8187898; doi:10.3389/fphar.2021.671572)

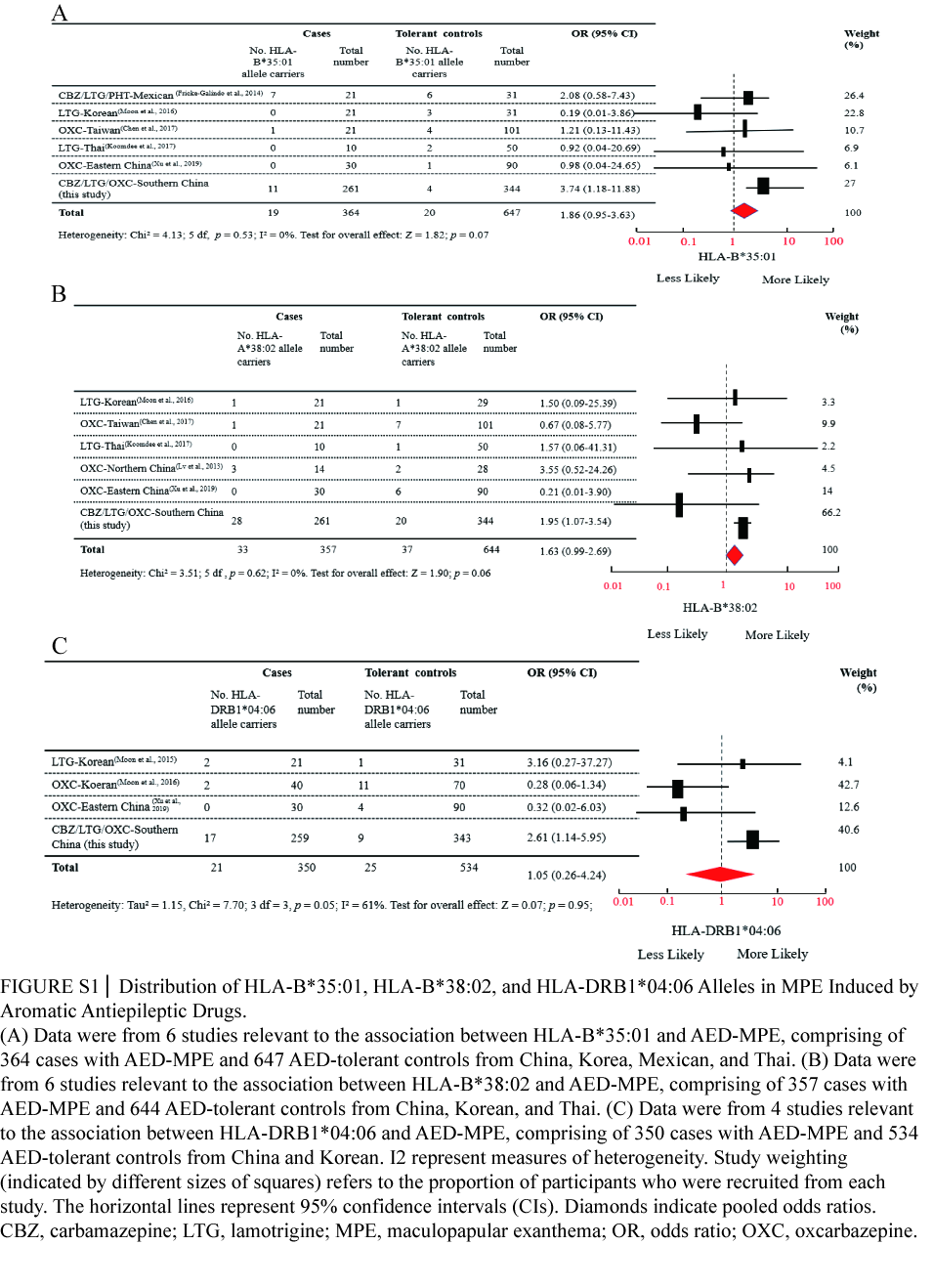

Supplement: Supplementary file 3 [file Image1.TIF]
